# Effect of Organic Carbon and Nitrogen on the Interactions of *Morchella* spp. and Bacteria Dispersing on Their Mycelium

**DOI:** 10.3389/fmicb.2019.00124

**Published:** 2019-03-01

**Authors:** Andrea Lohberger, Jorge E. Spangenberg, Yolanda Ventura, Saskia Bindschedler, Eric P. Verrecchia, Redouan Bshary, Pilar Junier

**Affiliations:** ^1^Laboratory of Microbiology, Institute of Biology, University of Neuchâtel, Neuchâtel, Switzerland; ^2^Laboratory of Biogeosciences, Institute of Earth Surface Dynamics, University of Lausanne, Lausanne, Switzerland; ^3^Stable Isotope and Organic Geochemistry Laboratories, Institute of Earth Surface Dynamics, University of Lausanne, Lausanne, Switzerland; ^4^Laboratory of Eco-ethology, Institute of Biology, University of Neuchâtel, Neuchâtel, Switzerland

**Keywords:** carbon and nitrogen, bacteria–fungi interaction, *Morchella* spp., soil, fungal highways, hydrolysis, fungal exudates, nutrient cycle

## Abstract

In this study we investigated how the source of organic carbon (C_org_) and nitrogen (N_org_) affects the interactions between fungi of the genus *Morchella* and bacteria dispersing along their hyphae (fungal highways; FH). We demonstrated that bacteria using FH increase the hydrolysis of an organic nitrogen source that only the fungus can degrade. Using purified fungal exudates, we found that this increased hydrolysis was due to bacteria enhancing the activity of proteolytic enzymes produced by the fungus. The same effect was shown for various fungal and bacterial strains. The effect of this enhanced proteolytic activity on bacterial and fungal biomass production varied accordingly to the source of C_org_ and N_org_ provided. An increase in biomass for both partners 5 days post-inoculation was only attained with a N_org_ source that the bacterium could not degrade and when additional C_org_ was present in the medium. In contrast, all other combinations yielded a decrease on biomass production in the co-cultures compared to individual growth. The coupled cycling of C_org_ and N_org_ is rarely considered when investigating the role of microbial activity on soil functioning. Our results show that cycling of these two elements can be related through cross-chemical reactions in independent, albeit interacting microbes. In this way, the composition of organic material could greatly alter nutrient turnover due to its effect on the outcome of interactions between fungi and bacteria that disperse on their mycelia.

## Introduction

Soils are living components of terrestrial ecosystems that sustain essential services such as nutrient recycling and productivity (Schnitzer and Klironomos, 2011; Baveye et al., 2016). The ability of soils to perform these services depends on both abiotic and biotic factors. Among the latter, fungi and bacteria are considered as central engines of soil nutrient cycling (Torsvik and Øvreås, 2002). However, the importance of other soil organisms such as protists, nematodes, and even viruses, in the cycling of nutrients in soils is also highlighted by recent functional studies (Adl and Gupta, 2006; Gebremikael et al., 2016; Geisen et al., 2016). In the carbon (C) cycle, fungi are known to degrade large polymers, including plant cell wall components such as cellulose, hemicellulose, and lignin (Kohler et al., 2015). In contrast, bacteria are active in the degradation of simpler polysaccharides (Lladó et al., 2016). Nitrogen (N) is a second important nutrient affecting soil ecosystem services (Kibblewhite et al., 2008; Mooshammer et al., 2014; Mooshammer et al., 2017). Apart of the mass production of inorganic N fertilizers in agriculture, the N cycle is virtually entirely controlled by prokaryotic microbes (Falkowski et al., 2008). Fixation of atmospheric nitrogen and the transformation of ammonium into nitrate, are reactions catalyzed only by prokaryotic enzymes that render N bioavailable to other organisms (Falkowski et al., 2008). In addition to nitrate and ammonium, which are considered the most abundant forms of bioavailable N in soils (Kant et al., 2010), dissolved organic N (e.g., small peptides and amino acids) represents a significant pool of N in many soils (Tamm, 1991; Jones et al., 2004; Hodge and Fitter, 2010; Hynson et al., 2015). Besides, more complex forms of organic N can contribute to the pool of available N for plant uptake through the interaction with symbiotic microorganisms such as mycorrhizae (Näsholm et al., 2009). While studies investigating the use of organic N are scarce (Horwath, 2017), the balance of C and N content in soils (C/N molar ratio) has been shown to affect the abundance of fungi and bacteria (Waring, 2013). This is because each element appears to influence one or the other microbial group (Demoling et al., 2007). Therefore, depending on availability of C and N, direct uptake or mineralization of organic C and N compounds (hereafter C_org_ and N_org_) can play an important role in determining soil microbial activity.

Beside the individual activity of fungi or bacteria in C and N cycling, a recent area of interest consists in investigating the role of bacteria–fungi interactions (BFI) in microbial activity (Frey-Klett et al., 2011; Deveau et al., 2018). Fungal colonization of terrestrial ecosystems likely had a strong impact on the evolution of bacteria, with soil bacteria living and evolving in a fungal world (Boer et al., 2005). The same can be expected reciprocally in the case of fungi. This long co-evolutionary history shapes nutrient cycling. Indeed, the classic fungi-only/bacteria-only view of nutrient turnover is now changing based on the recognition of BFIs occurring during organic matter mineralization, as reported, for instance, for lignocellulose degradation (Johnston et al., 2016) or wood cellulose and lignin degradation in forest soils (Valaskova et al., 2009; Hervé et al., 2016; Kielak et al., 2016; Rinta-Kanto et al., 2016). The same should be true in the mineralization of various N_org_ substrates, but this point has not been assessed so far.

Here, we focus on how one recently model system of BFI in soils, namely the active dispersal of bacteria on the surface of fungal mycelial networks, affects the mineralization of C_org_ and N_org_ compounds. This dispersal mechanism, dubbed “fungal highways” (FH) (Kohlmeier et al., 2005), expands the spatial range in which bacteria can be active, as bacterial mobility is otherwise limited by the discontinuity of water films in most soils (Kohlmeier et al., 2005; Dechesne et al., 2010; Wang and Or, 2010). FH have been suggested to be important in processes related to soil bioremediation (Wick et al., 2007; Furuno et al., 2010). In microcosm experiments, FH appears as one of the mechanisms explaining the role of fungi on activating the bacterial use of a specific C source (Martin et al., 2012; Bravo et al., 2013). In addition, recently, FH have been shown to affect the way microbial communities get assembled in a model ecosystem in which dispersal is highly limiting (rind cheese) (Zhang et al., 2015). The physical contact established between fungi and bacteria during FH opens up the possibility of a more direct nutrient exchange as well as cross-chemical reactions that may enhance or reduce microbial metabolic activity. In this study, we investigated the role of FH interactions established with different species of the fungal genus *Morchella* in the exploitation of C_org_ and N_org_ and their effect on fungal and bacterial biomass.

## Materials and Methods

### Description of the Fungal and Bacterial Species Used in the Study

Three fungal strains belonging to the genus *Morchella* (Ascomycota) were used in this study (Table 1). They were isolated from ascocarps collected in Switzerland. *Morchella crassipes* (NEU M84), *Morchella rufobrunnea* (NEU M85) and another isolate of *Morchella rufobrunnea* were identified by sequencing of the internal transcribed spacer (ITS) 1 and 2 and the 5.8S rRNA gene, *Morchella crassipes* is known to form FH with diverse bacteria (Pion et al., 2013a). Available information concerning the ecology of the genus suggest that *Morchella* spp. includes not only saprophytic species, but also facultative mycorrhizal species (Tietel and Masaphy, 2018). This fungal genus is of economic importance as the fruiting bodies of various species are used commercially as edible fungi and in traditional medicine (Tietel and Masaphy, 2018).

**Table 1:** Fungal and bacterial strains used in this study.

Kingdom/ Genus	Species	Tag	Motility	Natural habitant	Deletion mutant	Proteolytic activity	Optimal inoculation temperature °C	Origin/reference	Accession number
Fungi/ *Morchella*	*M. crassipes*	-	+	Soil	–	+	21°C	Soil isolate (Pion et al., 2013b)	JX258671.1
	*M. rufobrunnea*	–	+	Soil	–	+	21°C	Soil isolate	Sequence in SI
	*M. rufobrunnea* NEU M85	–	+	Soil	–	+	21°C	Soil isolate	Sequence in SI
Bacteria/ *Pseudomonas*	*P. putida* KT2440	Green fluorescent protein (GFP)	+	Soil	–	–	30°C	Lambertsen et al., 2004; Dechesne et al., 2010	AE015451.2
	*P. putida* KT2440 *ΔfliM*	Red fluorescent protein (DsRed)	–	–	Isogenic, non-flagellated mutant	–	30°C	Lambertsen et al., 2004; Dechesne et al., 2010	AE015451.2
	*P. fluorescens* CHAO *ΔgacA*	-	+	-	Isogenic mutant (deletion of *gacA,* a regulatory gene involved in motility and secondary metabolism, among others)	–	24°C	Laville et al., 1992	NR_043420.1
	*P. fluorescens* Pf0-1	–	+	+	–	–	30°C	Seaton et al., 2013	CP000094.2
	*P. fluorescens* Pf0 *ΔaprA*	–	+	-	Isogenic mutant lacking the *aprA* gene (alkaline protease)	–	30°C	Seaton et al., 2013	CP000094.2
	*P. aeruginosa* La 425	–	+	+	–	–	37°C		
	*P. azelaica* HBP1	–	–	Waste water treatment plant isolate		–	30°C	Kohler et al., 1988	FJ227303.1
Bacteria/ *Cupriavidus*	*C. necator* JMP 289	–	+	+	Plasmid-free and rifampicin-resistant derivative of *C. necator* JMP134	–	30°C	Sentchilo et al., 2009	EU827495.1
	*C. oxalaticus*	–	+	+		–	30°C	ATCC 11883	NR_025018.2
Bacteria/ *Escherichia*	*E. coli* K12	–	–	Water, lower gut of animals, vegetables, meat		–	37°C		CP025268.1


Seven strains belonging to the genus *Pseudomonas,* as well as two strains belonging to the genera *Cupriavidus* (two species) and *Escherichia* (one species) were included (Table 1). The specific characteristics of the strains, including the presence of fluorescent tags, motility, gene deletions, ecology (habitat), and proteolytic activity are shown in Table 1. *Pseudomonas putida* strain KT2440 and its isogenic non-flagellated mutant (strain *ΔfliM*) were kindly provided by Dr. Arnaud Dechesne (Technical University of Denmark). Both strains harbor in their chromosomes a gene coding for either a green fluorescent protein (GFP) or a red fluorescent protein (DsRed) in a neutral locus. These genes are expressed constitutively and were inserted in the chromosome with the miniTn7 transposon delivery plasmid (Lambertsen et al., 2004; Dechesne et al., 2010). The non-motile Δ*fliM* strain was constructed by allelic exchange (for details please refer to Dechesne et al., 2010). The deletion of gene *fliM* causes the absence of the flagellum (Dechesne et al., 2010) and makes *P. putida* KT2440 *ΔfliM* unable to disperse on FH (Pion et al., 2013b). According to the annotation of the genome of *P. putida* KT2440, this strain does not possess a casein-degrading protease (Supplementary Table S1). *Pseudomonas fluorescens* strain CHAO *ΔgacA* was constructed and kindly provided by the Laboratory of Prof. Dieter Haas (University of Lausanne). The strain carries a deletion on the gene *gacA* generated with a recombinant plasmid, which was constructed in cosmid pVK100 and mobilized with the helper plasmid pME497 by *Escherichia coli* to *P. fluorescens* CHAO (for details please refer to Laville et al., 1992). The gene *gacA* encodes a regulatory protein of the two-component system GacA/GacS, which controls the expression of secondary metabolites and extracellular products. Under the conditions tested here, this strain does not have a proteolytic activity. *P. fluorescence* Pf0-1 and Pf0 *ΔaprA* were kindly provided by the laboratory of Prof Stuart B. Levy (Tufts University School of Medicine, Boston). The gene *aprA* was deleted using the suicide vector pSR47S by amplification of the gene by PCR and interruption by the insertion in an antibiotic resistance cassette. The mutated alleles were finally cloned into the pSR47S plasmid and transferred by conjugation (for details please refer to Seaton et al., 2013). The *aprA* gene encodes for an alkaline protease, which is involved in proteolytic activity.

### Preparation of Inocula

A pre-inoculum of the fungal strains was prepared on malt agar medium (malt) (Supplementary Table S2) and incubated for 5 days at 21°C. The pre-inocula were made by placing a piece of malt previously colonized by mycelium on top of the fresh medium. The piece was cut using the large end of a sterilized Pasteur pipette. The inoculum was obtained from the outer rim of the colony.

A bacterial pre-inoculum was prepared from a cryopreserved culture by first re-culturing on nutrient agar (NA) medium (Supplementary Table S2) overnight. The cultures were incubated at the optimal temperate for each strain (24, 30, or 37°C as indicated in Table 1). This culture was then used to inoculate a fresh NA Petri dish as a bacterial loan. Pre-inocula were incubated overnight at optimal temperature for each individual species. To prepare the inoculum for the experiments, bacterial biomass was mechanically collected with a sterile spatula from the fully-covered NA plate. Biomass was transferred to an Eppendorf tube and washed three times in 1 mL saline aqueous solution (0.9% w/v NaCl) to avoid any carry over from the pre-inoculum medium. Between the washing steps, the bacterial inoculum was centrifuged at 5000 rpm for 5 min. After removing the medium, the inoculum was adjusted to the cell concentration of 10^8^ cells μL^-1^.

### Extracellular Proteolytic Activity

Proteolytic activity was tested on a skimmed milk medium with and without additional malt (malt/casein and casein, respectively; Supplementary Table S2). In the experiments with the fungal partner alone, a plug cut with the large end of a sterilized Pasteur pipette was obtained from the colony edge of the fungal pre-inoculum and placed in the center of a Petri dish containing the respective medium. The Petri dish was incubated at 21°C in darkness. In order to prepare co-cultures, the bacterial inoculum was added to Petri dishes with the fungus prepared as indicated before, after 1 day of incubation. The bacterial cells were added as a semicircle (5 μL) at around 1.5 cm from the fungal inoculum. Cultures with bacteria alone were prepared as above, but in the absence of the fungus. Six replicates were prepared for each condition. After adding the bacterial inoculum, images of the Petri dishes were taken 2, 3, and 5 days post inoculation (DPI), using a Canon Power Shot SX 230 HS digital camera. The close-up images of the colonies were taken with a Nikon C-BD230 stereomicroscope with different filters for bright field (Photonic PL3000 cold light as source; Nikon C-SHG1), GFP, and DsRed (mercury light source for fluorescence images; Nikon C-SHG1).

In order to compare the area of proteolysis from the fungal monocultures and bacterial-fungal co-cultures, the original 8-bit RGB images were analyzed using ImageJ (Version 2.0.0). The images were split into the three color channels (red, green, and blue). The blue channel, with the highest contrast, was used for further analysis. The picture was converted into a red image to adjust the threshold manually. After the image was converted into a black/white image, where the area of proteolysis appears black, a person naive to the experimental design manually selected the white zones within this area in black. The black area was then automatically surrounded with a selection tool and the extend of the area of proteolysis was analyzed in mm^2^ (*n* = 6). In order to test for significant outliers a Grubb’s Test was used (α = 0.01). A Shapiro–Wilk Test was then performed to test for normality of the data using a threshold of *p* = 0.01 for *n* = 6. The difference in the mean area of the proteolytic zone for each condition was then statistically compared using a two-tailed Students *T*-Test for 2 independent means with a threshold of *p* = 0.01.

### Extraction of Fungal Exudates

In order to control for the excess production of proteolytic enzymes by the fungus in presence of bacteria, fungal exudates were extracted and tested for proteolytic activity. Proteolytic activity was measured on exudates extracted from mono and co-cultures prepared on malt and malt/casein medium, as indicated in the respective figure legends. After 5 DPI, water-soluble fungal exudates were extracted by collecting the agar and transferring it to a 50 mL falcon tube. A 0.9% NaCl (w/v) solution was added until the 30 mL mark of the Falcon tube. This was incubated for 24 h, at 4°C. The supernatant was filter sterilized (0.2 μm nylon filter, Nalgene^®^). Exudates were also collected from specific areas of the fungal colony. For the latter, exudates were extracted from four different places of the fungal mono or co-culture by pouching out the agar with the tick end of a sterile Pasteur pipette. Three agar plugs from each place were placed in 200 μL of 0.9% NaCl (w/v) and incubated for 24 h at 4°C. The supernatant was filter sterilized as indicated before.

### Proteolytic Activity of Extracted Fungal Exudates in Presence and Absence of Bacteria

To test for proteolytic activity of the fungal exudates alone, 8 μL of the filter-sterilized fungal exudates were dropped on the surface of malt/casein agar (0.75% agar). To measure the effect of bacteria on proteolytic activity of the fungal exudates, bacterial cells were inoculated on NA as previously described. Bacterial biomass was recovered as described before (preparation of pre-inocula). Cells were then mixed with 30 mL of 40°C warm (non-polymerized) malt/casein agar medium (final bacterial concentration 5 × 10^7^ cells mL^-1^). The medium-bacterial mix was gently homogenized and poured into Petri dishes. Then, 8 μL of the filter-sterilized fungal exudates were dropped on the surface of the medium containing bacteria. To verify that enhancement depends on living bacterial cells, Petri dishes with three compartments of 10 mL each (no bacteria, living, and dead bacteria) were used for this purpose. Living bacteria were prepared and inoculated and as previously described. Dead bacteria were obtained by heating and freezing bacterial cells four times alternately for 5 min at 80°C in a water bath and 5 min at -196°C in liquid nitrogen. The control without bacteria contained only medium. On each compartment 8 μ filter-sterilized exudates were dropped on the surface (with the number of technical replicates indicated in the respective figure legends). Each combination was prepared in triplicate.

Pictures were taken 24 h after inoculation at 21°C with a Canon EOS 5D MK2 at same light and distance conditions (lens: Sigma f2.8/105 mm makro; exposure: camera raw, f8, 1/250 sek, adobe rgb). Images were processed in Adobe Photoshop from the raw images using the following settings: wb: 3900, color-tone: +45, contrast: +1; clarity +50. Quantification of the tonal value averages of each dropping spot was performed with ImageJ (Version 2.0.0). A circle of 30 pixels was placed in the middle of each spot and the average of the tonal value was analyzed. The Grubb’s Test was used to test for significant outliers. The Shapiro–Wilk Test was used to test for normality. A two-tailed Students *T*-Test for two independent means was also chosen to determine significant differences (*p* = 0.01) between the proteolytic activity of the fungal exudates in the presence and absence of bacteria.

### Effect of C_org_ and N_org_ Sources on the Outcome of *Morchella*–*Pseudomonas* Interaction

*Morchella crassipes* and *Pseudomonas putida* were selected to test the effect of varying C_org_ and N_org_ on biomass production. Pre-inocula were prepared as indicated previously. The fungus was then inoculated in the middle of a Petri dish (as previously described) on malt, malt/casein, casein, urea or malt/urea and incubated for 24 h at 21°C. After 1 DPI, 5 μL of a bacterial suspension (pre-inoculated bacteria as described before) were added at a concentration to 10^8^ cells μ^-1^. Both strains were inoculated as a mono or co-culture for 6 DPI. The experiments were performed in triplicates.

### Fatty Acid Analysis

The analysis of specific fatty acids (FA) was used to measure the effect of the different culture conditions (i.e., media type, mono versus co-culture) on the resulting *P. putida* and *M. crassipes* biomass. For this, quantitative analysis of specific phospholipid biomarkers for the bacterium and fungus was conducted (Olsson, 1999; Zelles, 1999). Phospholipid FAs were extracted from a total of 27 samples and were identified by gas chromatography/mass spectrometry (GC/MS). For quantification gas chromatography/flame ionization detection (GC/FID) was used (Spangenberg et al., 2014; Spangenberg, 2016). All chemicals [chromatography-grade solvents: dichloromethane, methanol, hexane, toluene; analytical grade (or higher) anhydrous sodium sulfate (Na_2_SO_4_) and 35% w/w hydrochloric acid (HCl)] were supplied by VWR/Merck International AG (Dietikon, Switzerland). Solvents were glass-distilled shortly before use. Fully deuterated lauric acid (D_23_*n*-C_12:0_) and arachidic acid (D_39_*n*-C_20:0_) were purchased from Cambridge Isotopes Laboratories (CIL, Tewksbury, MA, United States) and used for preparation of an internal standard solution in dichloromethane. Reagent-grade water was prepared by three liquid-liquid extractions of 500 m purified water using a Direct-Q UV 3 Millipore^®^ System (Merck, Darmstadt, Germany) with 50 mL dichloromethane.

For FAs analysis, Petri dishes containing medium and microbial biomass were frozen at -80°C for 2 days and then freeze-dried for 16 h. The medium and the microbial biomass were separated mechanically from the Petri dish using cleaned forceps and scissors and placed in a 12 mL borosilicate glass vials with Teflon-lines screw caps. An aliquot of internal standard solution containing a defined amount (50 mg) of deuterated carboxylic acids (D_23_*n*-C_12:0_, lauric acid, D_39_*n*-C_20:0_, arachidic acid) was added to each sample to allow downstream identification and quantification. FAs were then extracted by vortexing for 2 min and using sonication in solvents of decreasing polarity (10 min in 2 × 3 mL methanol, 10 min in 2 × 3 mL methanol/dichloromethane, 1:1, v/v; 10 min in 2 × 3 mL dichloromethane). The extracts were combined and the solvent removed via gentle evaporation under a clean nitrogen flow.

The extracted FAs were transesterified using the acid-catalyzed procedure to form fatty acid methyl esters (FAMEs) (Ichihara and Fukubayashi, 2010). The FA extract was dissolved in 0.20 mL toluene, and 1.5 mL methanol and 0.30 mL 8.0 vol. % HCl/methanol solution were added in that order. The mixture was vortexed for 1 min and incubated at room temperature for >16 h. After cooling to room temperature, 1.5 mL of hexane and 1.5 mL of reagent-grade water were added and the mixture vortexed for 1 min. After phase separation, the upper hexane layer, containing the FAMEs, was removed, dried with anhydrous Na_2_SO_4_, passed to a 2 mL vial with Teflon (PTFE)-lined screw cap, and stored at +4°C until analyses.

Chemical characterization of FAs was performed by GC/MS using an Agilent (Palo Alto, CA, United States) 6890 GC. The GC was connected to an Agilent 5973 mass selective detector operating at 70 eV (source 230°C and quadrupole 150°C) in the electron ionization mode with emission current of 1 mA and multiple-ion detection over *m/z* 10–450. Helium was used as carrier gas (1.4 mL/min flow rate). For the analyses of FAME fractions, the system was equipped with an Agilent free FA phase fused silica capillary column (50 m length, 0.20 mm i.d.) coated with nitroterephthalic acid modified polyethylene glycol stationary phase (film thickness 0.33 μm). A sample aliquot was injected split-less at a temperature of 200°C. Helium was used as carrier gas (1 mL/min flow rate). After an initial period of 2 min at 100°C, the column was heated to 240°C at 5°C/min followed by an isothermal period of 30 min. Compound assignment was based on comparison with standard mass spectra in the NIST14 Mass Spectral Library (National Institute of Standards and Technology, Gaithersburg, MD, United States), GC retention time, and MS fragmentation patterns. The FAs are abbreviated as x:y, where “x” is the number of carbons and “y” the number of double bonds. Concentrations of the FAs were determined by GC/FID using an Agilent Technologies (2850 Centerville Road, Wilmington, DE, United States) 7890B GC system equipped with a 7693A automated injection system and a flame ionization detector. The column and chromatographic conditions were the same used for GC/MS. Quantitative data were expressed in μg FA determined from the peak area ratios of unknown and internal standards of known concentrations. Quantification of phospholipids in the microbial cells were assessed with the total peak areas of the biomarker FAs cy17:0 for bacteria (Guckert et al., 1985) and 18:2w6 for fungi (Olsson, 1999; Zelles, 1999). These two biomarkers were selected based on a full comparison of the FAs profile of mono and co-cultures in different media (Supplementary Figure S1). The relative change of biomass was calculated according to the FA determination in μg/culture for each co-culture in comparison to the corresponding monoculture. To control for outliers, a Grubb’s Test was performed. After removing outliers, a student *t*-test with two-tail hypothesis was used with a significance at *p* < 0.05 to compare the change in relative biomass between mono and co-cultures.

## Results

### Proteolytic Activity by *Morchella crassipes* and Associated Bacteria

**FIGURE 1:**
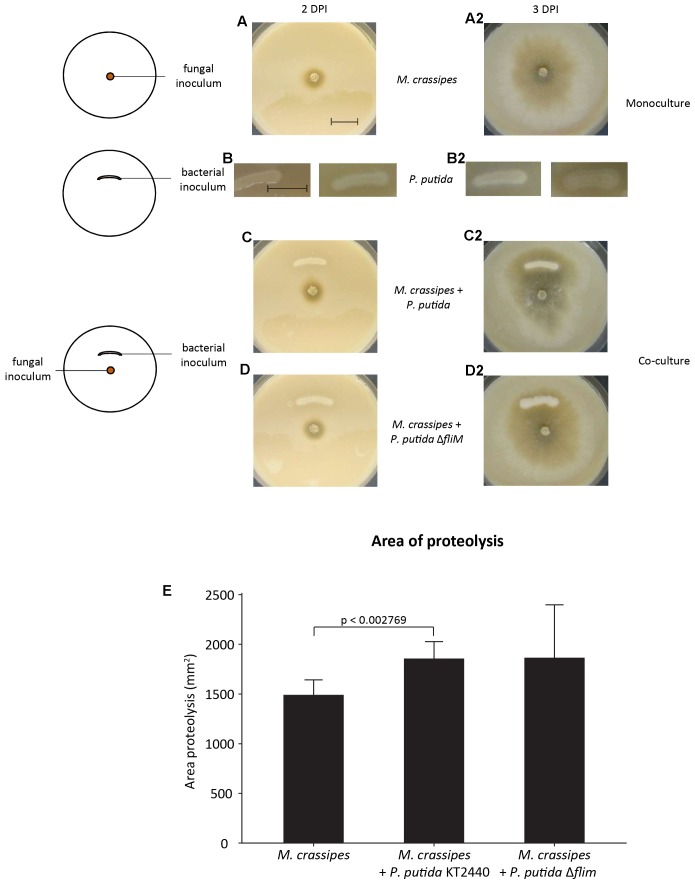
Extracellular proteolytic activity of *Morchella crassipes* in skimmed milk medium supplemented with malt as an additional carbon source. The experimental setup consisted of the inoculation of the fungus alone in the center of the Petri dish (left image, upper panel), the bacterial monoculture in form of a semi-circle (left image, center) or the two organisms together (co-cultures) with a bacterial inoculum at a fixed distance from the fungal inoculum (left image, lower panel). In this medium, proteolysis can be observed directly by the formation of a clearing halo around the growing mycelium after 2 **(A–D)** and 3 **(A2–D2)** days post-inoculation (DPI). Experiments were performed in mono-cultures of the fungus **(A,A2)** and bacterium **(B,B2)** or in co-cultures of the fungus with a motile strain of *Pseudomonas putida* KT2440 **(C,C2)** or its non-motile isogenic mutant (**D,D2**; *P. putida ΔfliM*). The experiment was performed in six replicates. After 2 DPI, no contact between the fungus and the bacterial inoculum was observed and accordingly, proteolysis was only observed around the fungal inoculum. The area of proteolysis was measured and compared for the different experimental conditions at 3 DPI, after contact with the bacterial inoculum. The difference in the proteolytic activity between the mono and co-culture with the two strains of *P. putida* KT2440 is statistically significant for the motile strain (**E**; *p*-value = 0.002769, *t*-value = –3.9414, two-tailed). A representative scale bar for the fungal mono and co-culture corresponding to 15 mm is shown in the first image.

Soil fungi are known to produce a large range of extracellular enzymes to access N from organic compounds (Leake and Read, 1990; Chalot and Brun, 1998; Read and Perez-Moreno, 2003; Nygren et al., 2007). However, the effect of FH on the hydrolysis of N_org_ compounds has not been evaluated so far. Proteolytic activity in a species of the genus *Morchella* (*M. crassipes*) was measured on skimmed milk (C/N molar ratio = 4.47) and malt (C/N = 55.84) medium (malt/casein medium) with milk proteins as N_org_ source and malt as an additional C_org_ source (Figure 1 and Supplementary Tables S2, S3). The fungus and the two *P. putida* strains (wild-type and non-motile mutant) were tested individually as well as in co-cultures. When tested as a monoculture, only the fungus showed proteolytic activity (Figure 1A,A2) but not the two bacterial strains (Figure 1B,B2). This confirmed the prediction made from the genomic information. In the fungal-bacterial co-cultures, proteolytic activity was higher than in the fungal monoculture (Figure 1C,C2). However, the results were only statistically significant for the co-culture with the motile *P. putida* strain, given the large variability in the results obtained with the non-motile strain (Figure 1E and Supplementary Figure S2). Thus, while proteolytic activity in malt/casein medium warrants the presence of fungal proteases, bacteria presence somehow enhanced the activity of these proteases.

**FIGURE 2:**
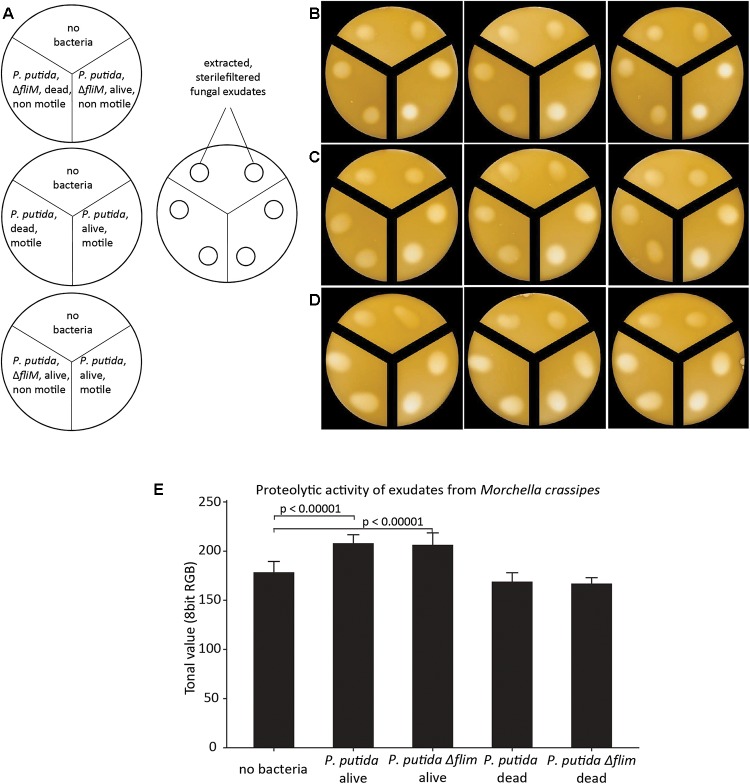
Effect of bacterial motility and viability on proteolytic activity of filter-sterilized fungal exudates measured on malt/casein medium after 24 h of incubation. The fungal exudates were extracted from a fungal mono-culture grown on malt 5 DPI. Proteolytic activity was measured in skimmed milk medium supplemented with malt (malt/casein). Schemes of experimental set up are shown in **(A)**. The three treatments investigated consisted of a control without bacteria, a dead bacterial culture of *P. putida* (motile or non-motile; *ΔfliM*) and corresponding living bacterial cultures. **(B–D)** Images of proteolytic activity of extracted fungal exudates dropped on malt/casein in compartmentalized Petri dishes, which corresponds to the experimental set-up indicated in **(A)**. The three panels represent biological triplicates. The images were analyzed with ImageJ by measuring the tonal value of the center of the proteolytic halo as averages of 8 bit RGB images in order to compare proteolytic activity in the different treatments **(E)**. In the graph only the *p*-values for the pair-wise comparisons between negative control (no bacteria) versus *P. putida* alive and no bacteria versus *P. putida ΔfliM* alive are indicated (*p* < 0.00001). The calculated *p*-values for the comparisons *P. putida* alive versus dead and *P. putida ΔfliM* alive versus dead were also *p* < 0.00001, but are not included in the graph. All *p* and *t* values were calculated with a student *t*-test, two-tailed. *T*-value for no bacteria/*P. putida* alive = –7.74455; no bacteria/*P. putida ΔfliM* alive = –6.64647; *P. putida* alive/dead = 8.43471; *P. putida ΔfliM* alive/dead = 7.10842.

The increase in fungal proteolytic activity might be either the result of fungal production of additional proteolytic enzymes in response to competition with bacteria for the uptake of Norg from hydrolysed compounds or due to bacteria enhancing the efficiency of the secreted fungal enzymes. We therefore tested the effect of bacteria on proteolytic activity of extracted fungal exudates, thus avoiding the production of additional proteases as an active response by *M. crassipes* to competition. We found that both *P. putida* strains were still able to enhance the activity of fungal proteases from purified fungal exudates obtained from cultures in different media and conditions (Supplementary Figure S3). This was measured by detecting not only the presence of a halo indicative of the proteolytic activity of the fungal enzyme, but also the increase in brightness of the proteolysis spot as compared to the control without bacteria (Figure 2A–D; upper compartment) or with dead bacterial cells (Figure 2A–C; left compartment). When the effect of living bacteria (Figure 2A–C; right compartment; Figure 2D; left and right compartment) versus dead bacteria (Figure 2A–C; left compartment) was compared (Figure 2A–D), the results showed that dead bacteria have no effect on the proteolytic activity, while living *P. putida* cells (regardless of the presence of the flagellum) are required to significantly stimulate the proteolytic activity of *M. crassipes* exudates (Figure 2E). In conclusion, these results show that living bacteria enhance fungal proteolytic activity, and this on itself could explain the results without considering the need for an increase in the production of proteolytic enzymes by the fungus as the result of competition.

### Enhanced Proteolysis by Bacteria in Other *Morchella* Species

In order to test if the results obtained in the case of *M. crassipes* correspond to a physiological property extended to other species in the same genus, two additional *Morchella rufobrunnea* strains, as well as different bacterial species were also studied (Table 1). The positive effect of both *P. putida* strains on the proteolytic activity of fungal exudates was also observed for exudates extracted from the two additional *Morchella* strains (Supplementary Figure S4). For these fungal strains, we confirmed a statistically significant effect of living bacteria on proteolytic activity (Supplementary Figure S5). Different bacterial strains, able or not to use FH, were also tested with *M. crassipes*. Enhancement of the proteolytic activity of *M. crassipes* exudates was observed for all the bacteria dispersing using FH, which included most *Pseudomonas* spp. and two *Cupriavidus* species. Bacteria that do not disperse naturally on fungal mycelium (in opposition to the artificially non-dispersing flagellar mutant), which included *P. azelaica* HBP1 (isolated from a wastewater treatment plant) and *E. coli* K12, had no effect on proteolytic activity of *M. crassipes* exudates (Supplementary Figure S6). The exact mechanism of enzymatic enhancement is still unknown but appears to be common in bacteria from different taxonomic clades able to establish FH.

### Effect of C_org_ and N_org_ Sources on the Biomass of *M. crassipes* and *P. putida*

**FIGURE 3:**
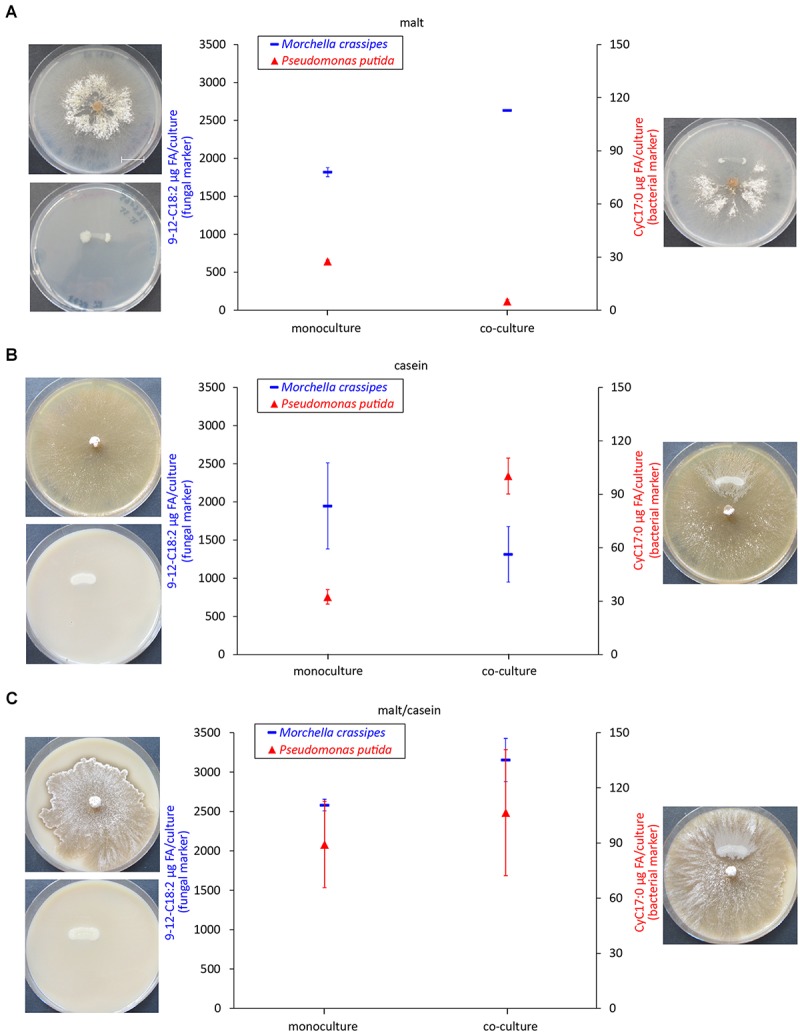
Biomass quantification as a proxy of bacterial and fungal fitness. Measured levels of specific fatty acid methyl esters (FAMEs) for the fungus *M. crassipes* and *P. putida* in monocultures (left point) and as a co-culture (right point). The experiment was performed in triplicates. A representative images of the corresponding cultures is shown on the side of each graph. The media used consisted of malt agar (**A**; malt), skimmed milk agar (**B**; casein), and skimmed milk agar supplemented with malt (**C**; malt/casein). *Y*-axis (left): Fungal Biomarker (red), a *n*-alkadienoic acid, 9-12C18:2 linoleic acid; *y*-axis (right): Bacterial biomarker (blue), a cyclopropane alkanioc acid, Cy17:0. A representative scale bar, indicating 15 mm, is shown in **(A)**. To control for outliers, a Grubb’s Test was performed. After removing one outlier for the fungal mono- and co-culture in malt, a *t-*test was performed for each medium condition for the fungal and bacterial mono- vs. co-culture. For the statistical analysis, the student *t*-test with two-tail hypothesis was used with a significance at *p* < 0.05. The *t*-value for the bacterial mono- vs. co-culture in malt is 28.04923. The *p*-value is *p* < 0.00001. The *t*-value for the bacterial mono- vs. co-culture in casein is –10.7964. The *p*-value is 0.0004. The *t*-value for the fungal mono- vs. co-culture in casein is 1.63832. The *p*-value is 0.176697. The *t*-value for the bacterial mono- vs. co-culture in malt/casein is –0.72416. The *p*-value is 0.509055. The *t*-value for the fungal mono- vs. co-culture in malt/casein is –3.49871. The *p*-value is 0.0249.

An increased proteolytic activity does not allow conclusions about the actual N uptake and who benefits of the increase in fungal proteolytic activity. Most studies on FH have focused on the benefits for the bacterial partner by quantifying cells and surface area colonized (Pion et al., 2013b). As it stands, proper fitness measures are very difficult to obtain for fungi, and BFI studies face the additional challenge to separate the individual effect on the fungal and bacterial partners. Therefore, we selected our best-studied pair (*M. crassipes* and the flagellated strain of *P. putida*) and measured specific phospholipid biomarkers, as an indirect proxy to infer the effect of hydrolysis of different C_org_ and N_org_ sources on biomass production. A change in biomass can be measured indirectly by the quantitative analysis of specific phospholipids extracted from mono and co-cultures (Olsson, 1999; Zelles, 1999). An inferred increase in the biomass of both partners in co-cultures relative to monocultures would provide evidence for increased C_org_ and N_org_ turnover as a result of enhancement of fungal proteases by dispersing bacteria. A unilateral inferred increase in biomass (or no increase) would provide evidence for competition between the two microbial partners. We found different responses in inferred relative biomass changes between different media. In a medium containing malt only, inferred bacterial biomass significantly decreased (*p* < 0.00001) in the co-culture (Figure 3A and Supplementary Figure S1). In contrast inferred fungal biomass was higher in the co-culture as compared to the monoculture, but the statistical significance of the results could not be assessed (Figure 3A). Nonetheless, these results agree with a previous study that has documented the use of bacterial biomass as a nutrient source (bacterial farming) by *M. crassipes* in this medium (Pion et al., 2013b). Accordingly, our results suggest that the fungus benefits in terms of biomass production in the harvesting phase of farming by using *P. putida* cells as an additional nutritional source. In a medium with casein but without malt as an additional C_org_ source, inferred bacterial biomass was significantly higher (*p*-value = 0.0004) in the co-culture while fungal biomass decreased as compared to the monoculture but this was not statistically significant (Figure 3B). In contrast, in malt/casein medium, both inferred fungal and bacterial biomass was higher in the co-cultures as compared to the monocultures, but in both cases the difference is not statistically significant (Figure 3C). Interestingly, bacterial farming was never triggered in either of the media supplemented with skimmed milk (Supplementary Figures S7, S8), indicating that a reduced content in N_org_ is likely a key factor triggering bacterial farming in the case of *M. crassipes*.

**FIGURE 4:**
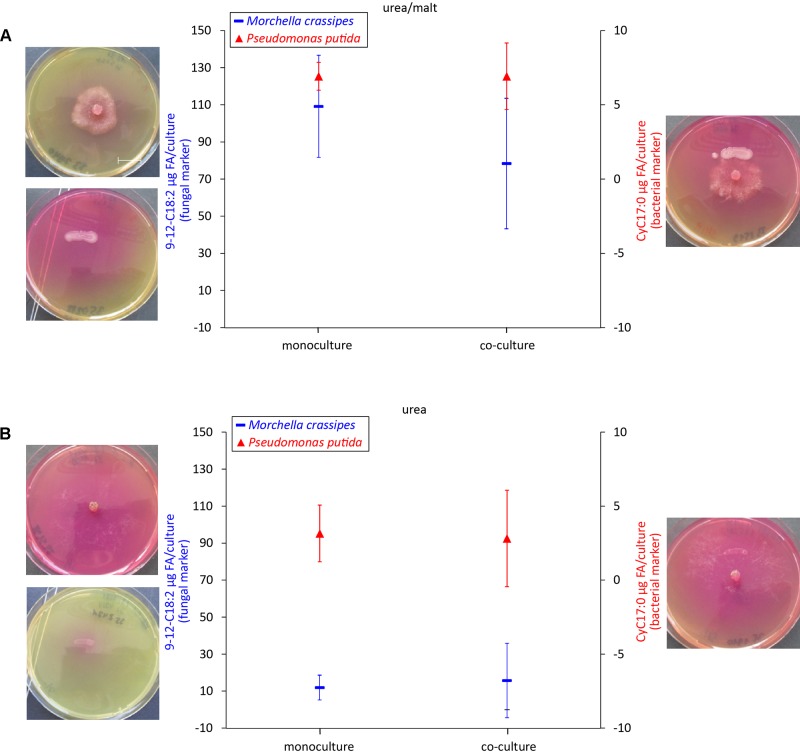
Biomass quantification as a proxy of bacterial and fungal fitness using urea as an alternative N_org_ source. Measured levels of specific fatty acid methyl esters (FAMEs) for the fungus *M. crassipes* and *P. putida* in mono-cultures (left point) and as a co-culture (right point). The experiment was performed in triplicates. Images of the corresponding cultures are shown on each side of the graph. The media used consisted of urea agar supplemented with malt (**A**; malt/urea), and urea agar (**B**; urea). *Y*-axis (left): Fungal Biomarker (red), a *n*-alkadienoic acid, 9-12C18:2 linoleic acid; *y*-axis (right): Bacterial biomarker (blue), a cyclopropane alkanioc acid, Cy17:0. A representative scale bar, indicating 15 mm, is shown in **(A)**. Grubb’s Test was performed to sort out outliers but no outliers were detected. For the statistical analysis, the student *t*-test with two-tail hypothesis was used with a significance at *p* < 0.05. The *t*-value for the bacterial mono- vs. co-culture in urea/malt is 0.02162. The *p*-value is 0.983788. For the media-conditions in urea/malt, the *t*-value for the fungal mono- vs. co-culture is 1.19366. The *p*-value is 0.2985. The *t*-value for the bacteria mono- vs. co-culture in urea is 0.15578. The *p*-value is 0.883754. The *t*-value for the fungal mono- vs. co-culture in urea is –0.31471. The *p*-value is 0.7687.

In order to test the effect of the N_org_ source on the interaction, in addition to skimmed milk we investigated urea as a second relevant N_org_ source, as urea is commonly found in soils. Moreover, urea (C/N = 0.5, Supplementary Table S3) is a N_org_ compound readily used as N fertilizer. In contrast to casein, for which only the fungus possesses proteolytic activity, both the bacterium and the fungus produced ureases to degrade this N_org_ source. However, in the case of the bacterium, ureases could only be produced in medium supplemented with malt as an additional C_org_ source (Figure 4). In medium containing both, urea and malt, inferred bacterial biomass remained unchanged, and fungal inferred biomass decreased, but this was not statistically significant (Figure 4A). In this condition, no FH were established as the fungus avoided contact with the bacterial inoculum (Supplementary Figure S9). In medium containing urea alone, the changes in biomass for either microbial partner were not significant (Figure 4B). Supplementary Figure S10 summarizes the highly variable mean net effects of C_org_ and N_org_ sources on *M. crassipes* and *P. putida* biomass change in co-culture.

## Discussion

In this study, we provide evidence for a cross-chemical reaction involving fungi of the genus *Morchella* and bacteria. This reaction corresponded to the hydrolysis of a N_org_ source in the form of proteins, more specifically those found in skimmed milk. We show a cross-chemical reaction in the case of proteases secreted by three strains of two species of the fungal genus *Morchella*. This effect was shown for bacteria establishing different types of interactions with the fungus, not only considering those that are able to disperse using FH, but also some that act as fungal biocontrol agents (e.g., *P. aeruginosa* CHAO). In addition, the enhancement of *Morchella* spp. proteolytic activity is unlikely the consequence of direct fungal competition with bacteria, as the effect was maintained for purified fungal exudates.

The exact mechanism by which bacteria enhance proteolytic activity remains unknown. However, given the nature of the enzymes involved (proteases), some hypothesis can be proposed. Extracellular digestion of protein substrates is required for survival and growth of all fungi (Yike, 2011). Casein-degrading proteases have been characterized in a variety of fungal species. These enzymes appear to belong to several classes of peptidases (da Silva, 2017), and to be widespread across different species (de Souza et al., 2015). Their optimal reaction conditions vary greatly in terms of pH (from acid to alkaline) and temperature (for example, from 28°C in the case of *Humicola lutea* to 70°C for *Thermomyces lanuginosus*) (de Souza et al., 2015). Potential mechanisms that could explain the activity observed are the modification of the local pH by bacterial growth (Scanlon et al., 2018), the effect of bacteria on the availability of an enzymatic co-factor (e.g., metals; (Riordan, 1977)), or the secretion of a specific co-factor by bacteria. Nevertheless, for advancing in the mechanistic understanding of the process, the purification and characterization of the enzyme(s) involved is required. This could not be achieved here. Fungal proteases are very important hydrolytic enzymes that had attracted considerable attention given their biotechnological use (de Souza et al., 2015) and their role in pathogenesis (Yike, 2011; Han et al., 2017). Understanding how BFI modulate the activity of this type of enzymes could therefore improve our understanding of these processes.

The results also show that different N_org_ sources, the presence of an additional C_org_ source, as well as the simultaneous presence of enzymes in the interacting couple, result in a variable outcome of the interaction in terms of the potential benefit in growth for each partner. This variable effect on hydrolysis of N_org_-containing compounds during bacterial dispersal on the growth of either (or both) the fungal and/or the bacterial partner offers a new perspective on FH, besides the purely mechanical aspect of bacterial dispersal on a fungal support. An exchange in nutrients has been postulated as explaining the interaction of fungi and bacteria living inside fungal hyphae (Frey-Klett et al., 2011; Ghignone et al., 2012). Likewise, similar processes of nutritional complementation and interference appear to play a role in the association of fungi with extracellular bacteria using FH. Given the fact that FH appear to be widespread and involve a large diversity of bacterial and fungal clades (Kohlmeier et al., 2005; Wick et al., 2007; Banitz et al., 2011; Warmink et al., 2011; Simon et al., 2015, 2017), future studies should investigate additional interacting species (e.g., a range of fungi and bacteria with different ecologies) and metabolic processes potentially modified by BFIs.

Fungal highways and the cross-chemical reactions established between *Morchella* spp. and bacteria associated in this dispersal mechanism can be instrumental to a better understanding of soil nutrient cycling. Such exchanges between fungi and bacteria may be essential in C and N cycling in soils and other ecosystems in which this interaction is expected to be one of the occurring BFI. Moreover, the enhanced access to C_org_ and N_org_ associated to FH can have an impact at higher trophic levels. For instance, N availability plays a key role in CO_2_ uptake by plants (Terrer et al., 2016). In the case of ectomycorrhizal fungi, the ability to access N_org_ through secreted proteases has been related to their ecological niche differentiation as part of the natural forest succession (Rineau et al., 2016). For example, in temperate and boreal forest, N is the element limiting tree nutrition (Rees et al., 2001). This fosters symbiosis with mycorrhizal fungi (Chalot and Brun, 1998) and shoot-endophytic bacteria (Carrell and Frank, 2014). Under these conditions, a bypass of nutrient acquisition directly from organic sources might be an advantage for plants associated to fungi. Indeed, plants associated with mycorrhizal fungi (largely providing N from organic sources) showed up to 30% biomass increase in response to elevated CO_2_ (Talbot and Treseder, 2010; Whiteside et al., 2012; Terrer et al., 2016; Norby et al., 2017). Our findings suggest that, through the exploitation of N_org_, not only mycorrhizal fungi, but also bacteria dispersing on their mycelial network, could play an important role in supporting plant CO_2_ uptake (Ainsworth and Rogers, 2007; Zhu et al., 2016). The interaction of fungi and bacteria could help plants associated to mycorrhizal fungi to better access N from complex organic sources such as proteins, and thus improve atmospheric CO_2_ fixation in plant biomass. Recently, it has been shown that C supply of the host plant can trigger the uptake and transport of N in the symbiosis with arbuscular mycorrhizal fungi (Fellbaum et al., 2012). Similarly, ectomycorrhizal fungi can enhance the mobilization of N_org_ from decomposed soil organic matter as a result of C supply from the plant, especially in boreal forest ecosystems (Lindahl and Tunlid, 2015). Enhanced growth of plants could lead to a positive feedback by providing more C to the soil as litter (either below- or above ground), which is in turn needed for the synergistic enhancement of proteolytic activity observed here in FH interactions. Therefore, a plant in association with a fungus having enhanced access to complex N_org_ due to dispersing bacteria could also benefit throughout this BFI. In addition, another potential implication of the interaction observed in this study is the negative impact of some agricultural practices that alter the soil fungal and bacterial network [for example, tillage (Beare et al., 1997; Zhang et al., 2016; Sharma-Poudyal et al., 2017)] on the efficiency of the recycling of C_org_ and N_org_ in soils.

## Author Contributions

AL conducted the experiments. JS quantified biomass together with AL. YV conducted the experiments. SB and EV supervised the project. RB conceived the experiments and supervised the analysis of the results. PJ conceived and supervised the project. All the authors wrote the paper.

## Conflict of Interest Statement

The authors declare that the research was conducted in the absence of any commercial or financial relationships that could be construed as a potential conflict of interest.
