# Supplementary material for: Effect of Organic Carbon and Nitrogen on the Interactions of Morchella spp. and Bacteria Dispersing on Their Mycelium
Source: Front Microbiol. 2019 Mar 1;10:124. doi: 10.3389/fmicb.2019.00124 (PMC6405442; doi:10.3389/fmicb.2019.00124)
Supplement: Supplementary file 1 [file Data_Sheet_1.pdf]

Supplementary information

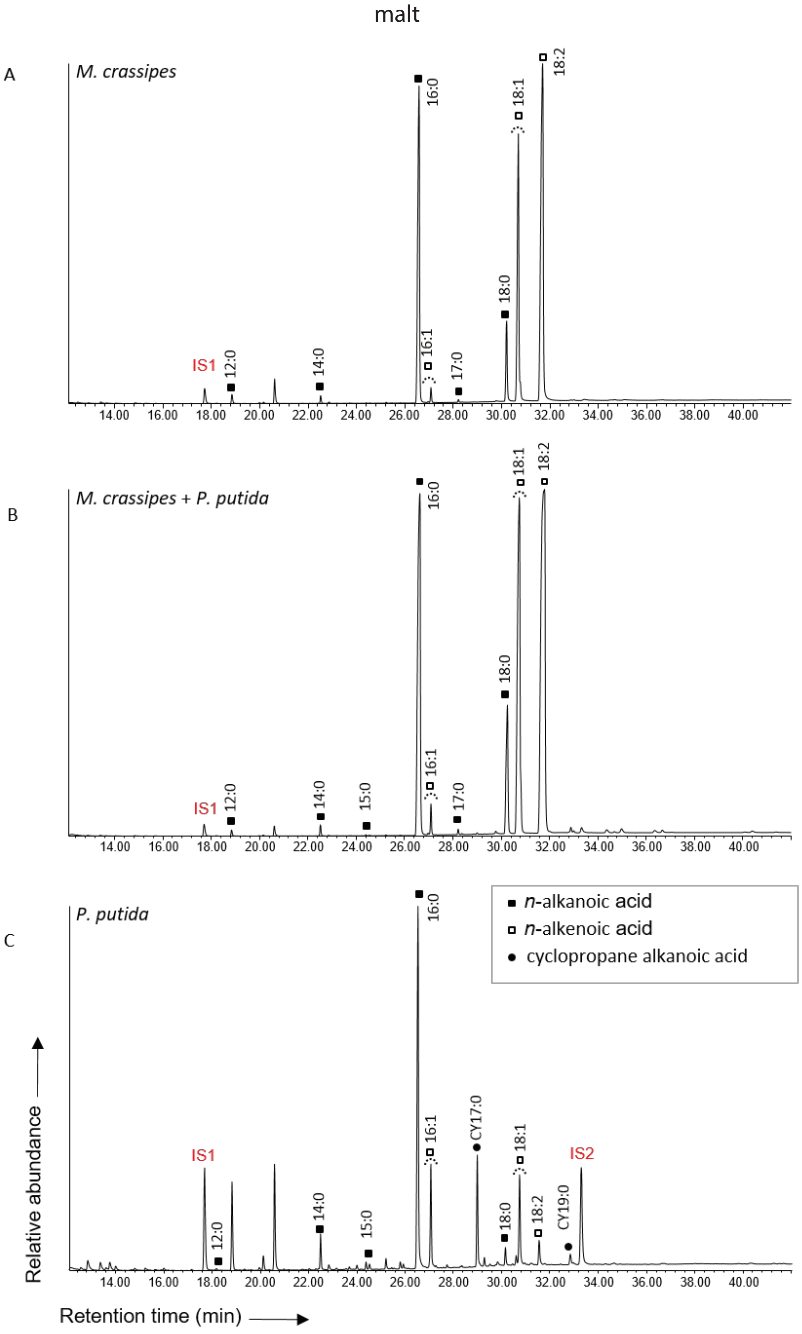

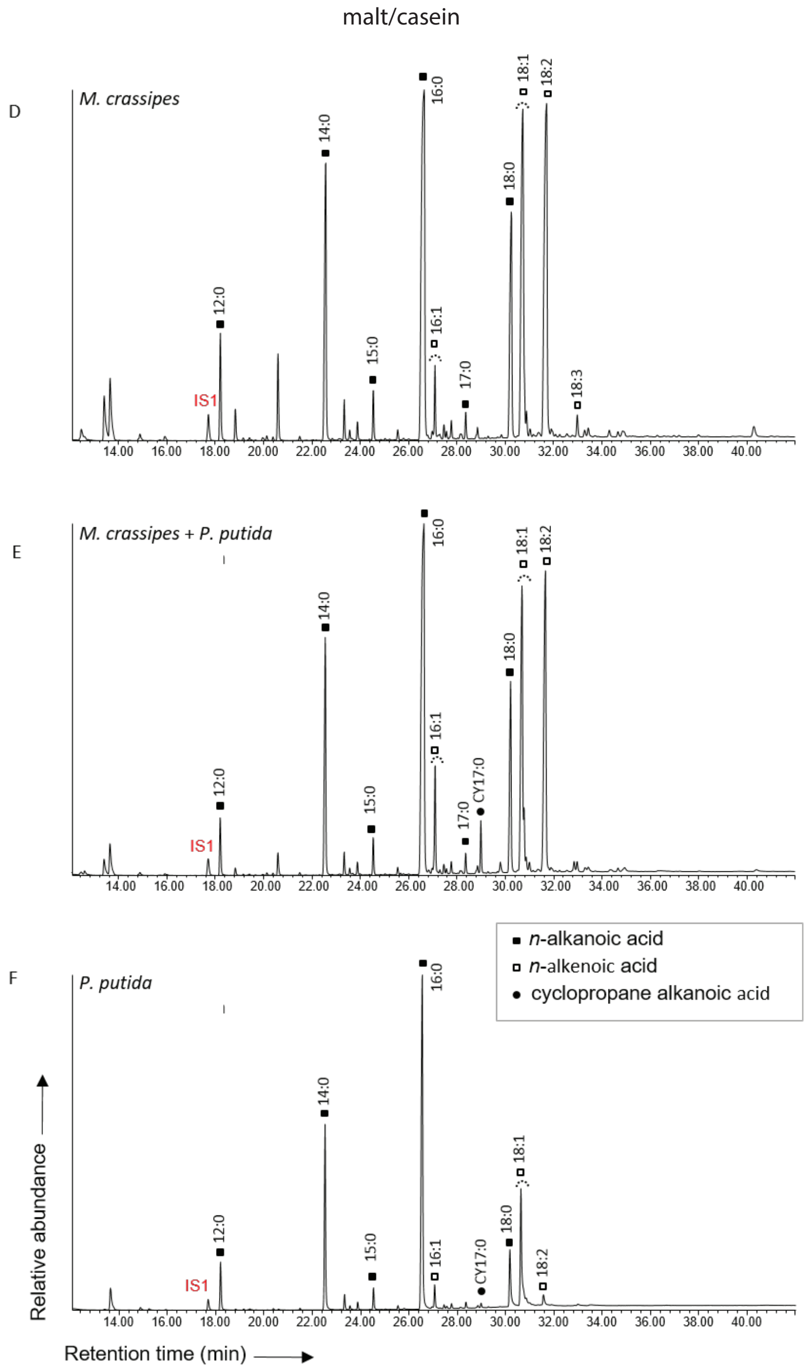
malt/casein
D
M. crassipes
E
M. crassipes + P. putida
F
P. putida
IS1
12:0
14:0
15:0
16:0
16:1
17:0
18:0
18:1
18:2
18:3
CY17:0
n-alkanoic acid
n-alkenoic acid
cyclopropane alkanoic acid
Relative abundance
Retention time (min)
14.00
16.00
18.00
20.00
22.00
24.00
26.00
28.00
30.00
32.00
34.00
36.00
38.00
40.00

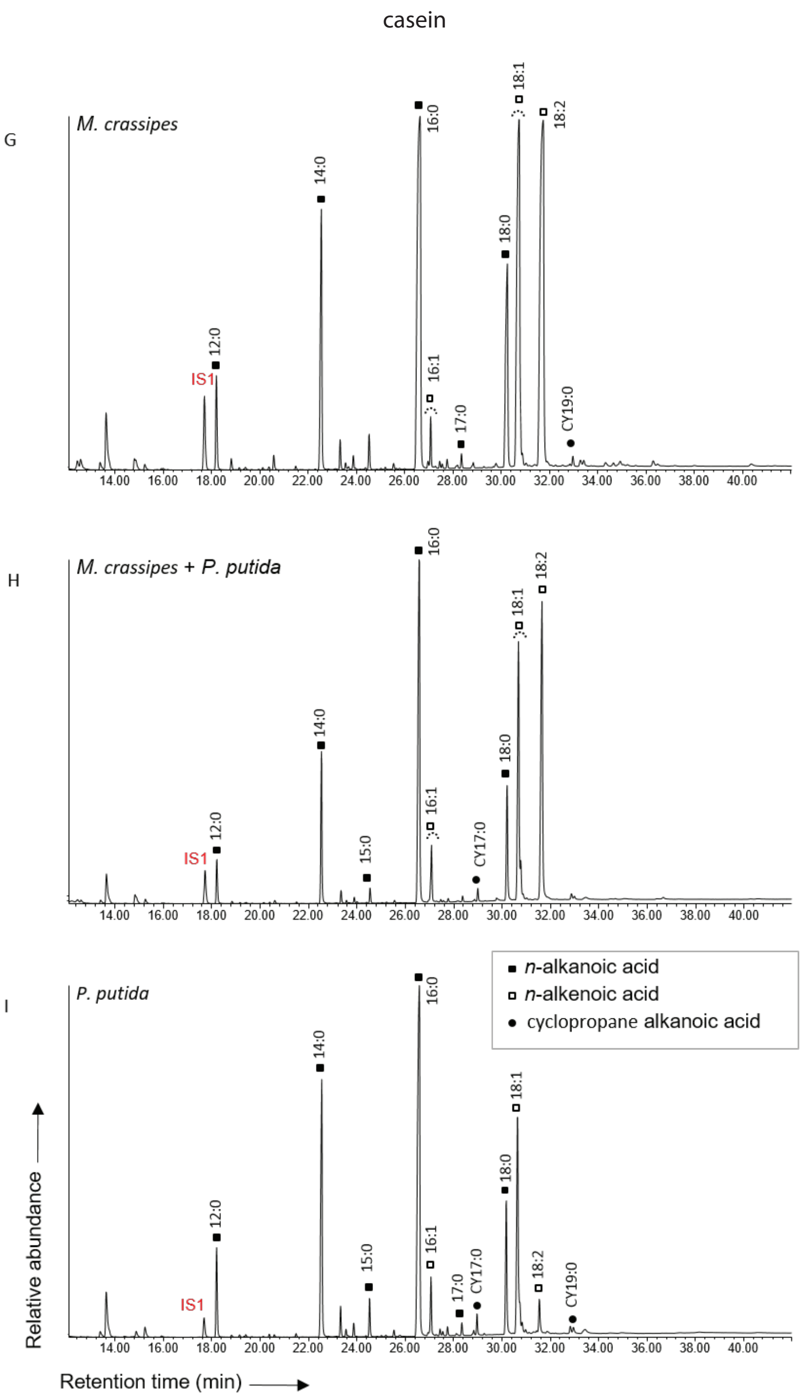

Figure S1. GC/MS chromatograms of the fatty acid methyl esters of the different cultures and conditions used in the experiments. The main fatty acids are the lauric (12:0), myristic (14:0), pentadecanoic (15:0), palmitic (16:0), palmitoleic (16:1), margaric (17:0), 2-hexyl-cyclopentaoctanoic (cy17:0), stearic (18:0), linoleic (18:2), 2-octyl-cycloprapenoctanoic (cy19:0), and linolenic (18:3) acids. The internal standards (IS) were deuterated lauric acid (D23-12:0) and deuterated arachidic acid (D39-20:0). The relative abundance of bacterial to fungal biomass was assessed from the concentration ratios of their fatty acid biomarkers (cy17:0 for bacteria and 18:2 for fungi).

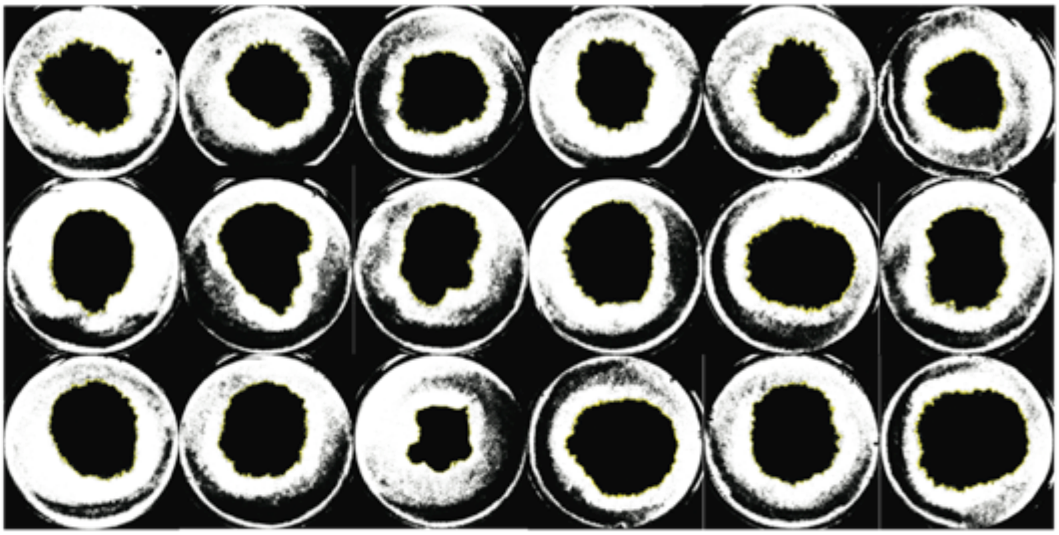

Figure S2. Analysis of the proteolytic activity on skimmed milk-agar medium supplemented with malt (malt/casein) three days post-inoculation (3 DPI). Images showing the proteolytic halo of cultures containing *Morchella crassipes* in monoculture (upper line) or in co-culture with a motile *Pseudomonas putida* KT2440 (middle line) or its isogenic non-flagellated mutant (Δ*fliM* (base line)) of *P. putida*. Original 8-bit RGB images were analyzed with ImageJ (Version 2.0.0) by splitting them into channels. The blue channel with the highest contrast was used for analysis. The threshold was adjusted manually and the pictures were converted into a black/white image. The black area, which indicates the proteolytic zone, was manually surrounded with a selection tool (indicated in yellow) and the area was then analyzed for all monoculture and co-cultures (n = 6). The selection of the area was done by a person naïve to the experimental conditions under comparison using randomly shuffled images of the three experimental conditions. The mean of each condition was then statistically analyzed. The results are shown in Figure 1E.

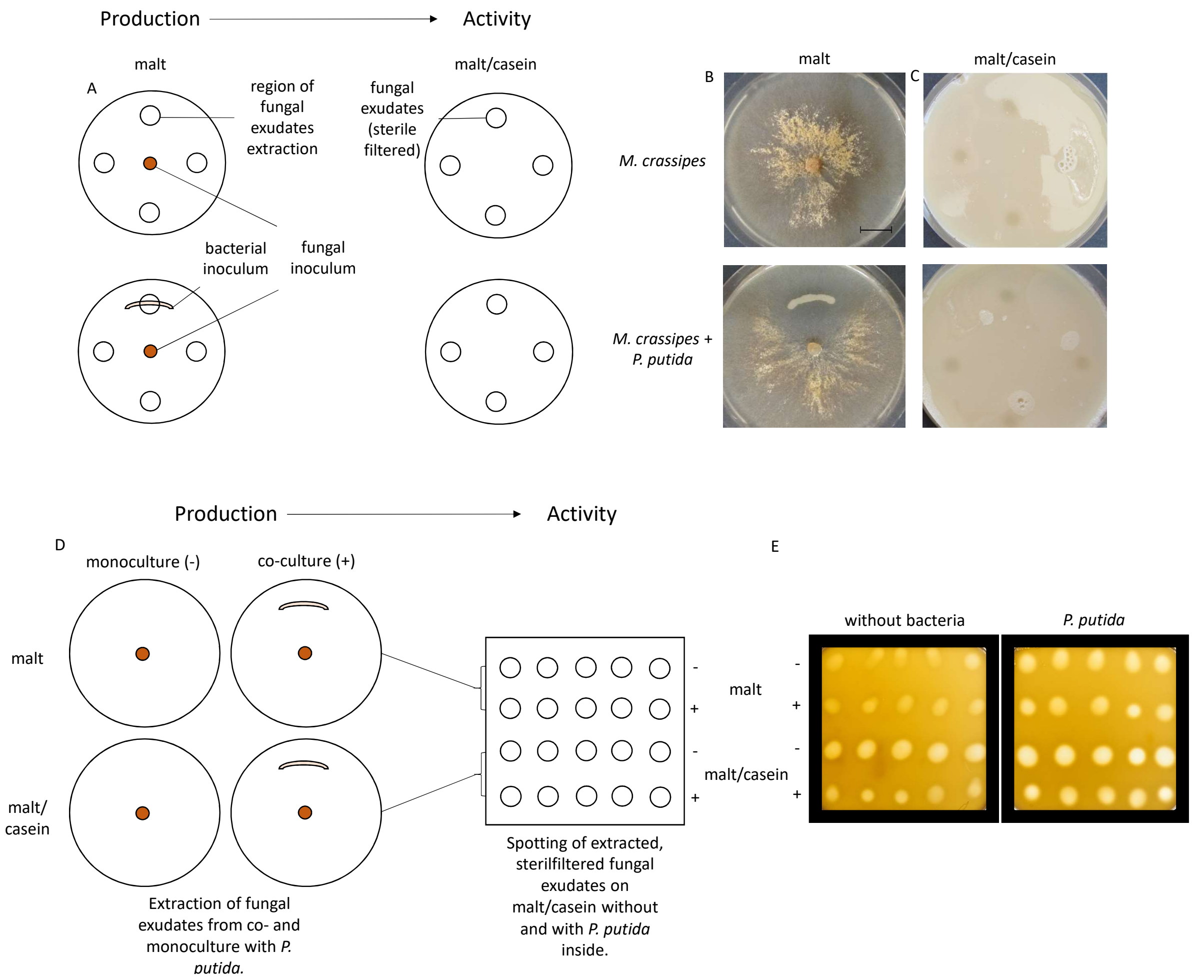

Figure S3. Extracellular production of proteolytic enzymes by *Morchella crassipes* and independent assessment of the enhancement of fungal proteolytic activity by *Pseudomonas putida*. To exclude any bias due to heterogeneous production of proteolytic fungal exudates in different areas of the mycelium or in response to contact with bacteria, *M. crassipes* was inoculated both in mono- or co-cultures on two media: skimmed milk-medium with malt (malt/casein) and malt-only (malt). Exudates were extracted from several places in the plates, as indicated in the scheme shown in **A** and the plates in **B**. Proteolytic activity of the extracted exudates was measured by dropping a fix volume of the extracts onto malt/casein without and with *P. putida* homogenously mixed within the malt/casein. No difference in the proteolytic activity was observed for the spotted volume of the filter-sterilised exudates, regardless of the position of the extract or the presence of bacteria (**C**). However, when the activity from exudates extracted from fungal cultures growing in the two media was compared (**D**), an increase of activity was observed when fungal cells were pre-exposed to N_{org} during growth (5 technical replicates for each condition; **E**). Fungal exudates extracted from malt-medium in monoculture are indicated with malt (-), extracted from co-culture are indicated with malt (+). The same labelling is used for fungal exudates extracted from malt/casein.

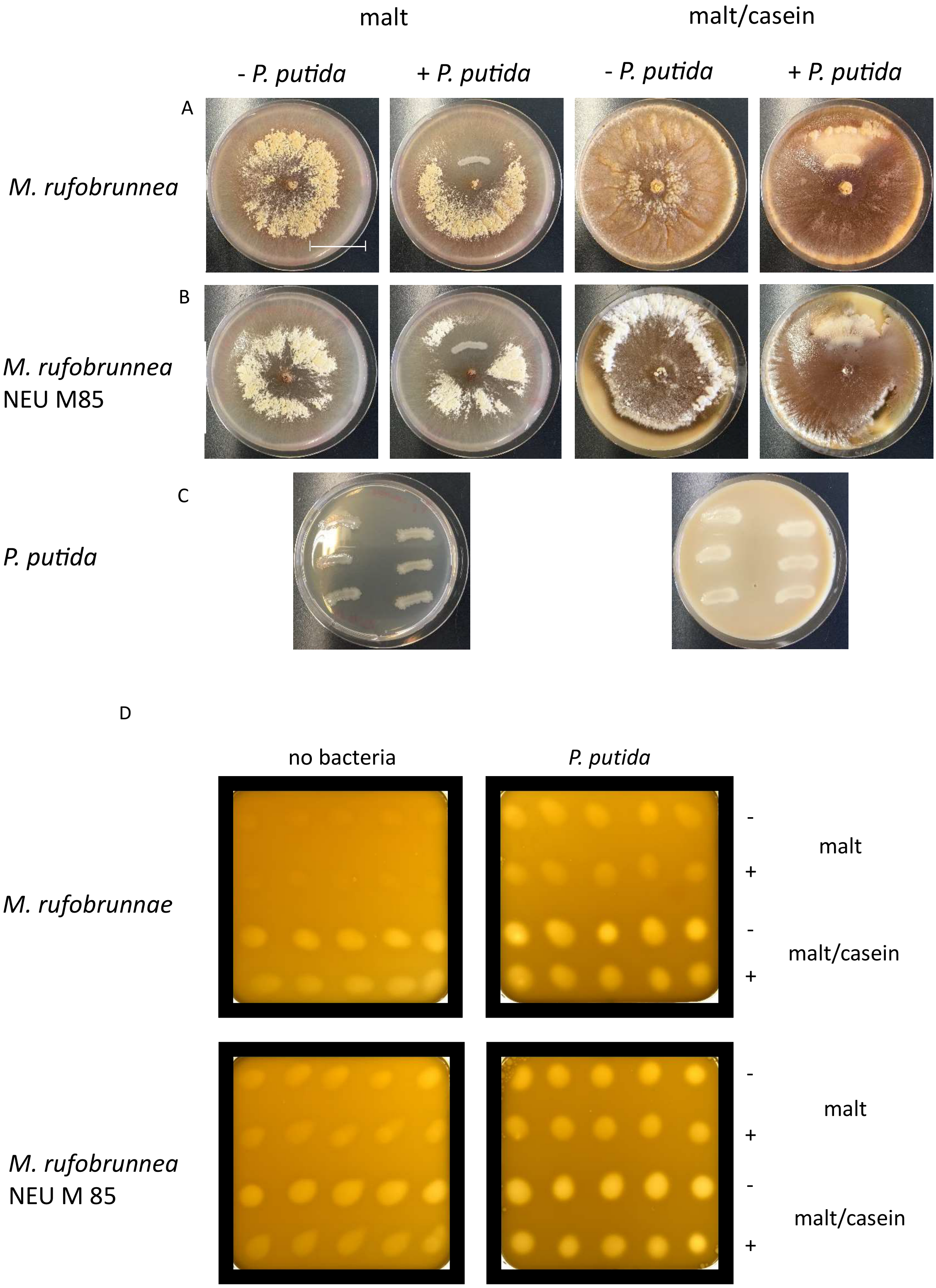

Figure S4. Production of extracellular fungal exudates in *Morchella rufobrunnea* (A) and *M. rufobrunnea* NEU M85 (B) in malt medium and skimmed milk-agar medium supplemented with malt (malt/casein) in mono- and co-cultures with *Pseudomonas putida*. The experimental setup consisted of the inoculation of the fungus alone in the centre of the Petri dish (first and third column) or together with an inoculum of the motile strain of *P. putida* KT2440 at a fixed distance from the fungal inoculum as a semicircle line (second and fourth

column). In A and B, the first two columns show the cultures on malt-medium (malt). Columns three and four show the cultures on malt/casein. Proteolysis can be seen by the formation of a clearing halo around the growing mycelium (here six days post-inoculation). A bacterial control for proteolytic activity was performed on both media and shown in **C.** A representative scale bar in A corresponds to 15 mm. **D.** The proteolytic activity of the extracted exudates was assessed dropping a fix volume of the extracts onto malt/casein in absence of bacterial cells (left panels) or presence of bacterial cells homogenously mixed in the agar (right panels). Fungal exudates extracted from malt-medium in mono-culture are indicated with malt (-), extracted from co-culture are indicated with malt (+). The same labelling is used for fungal exudates extracted from malt/casein (5 technical replicates for each condition).

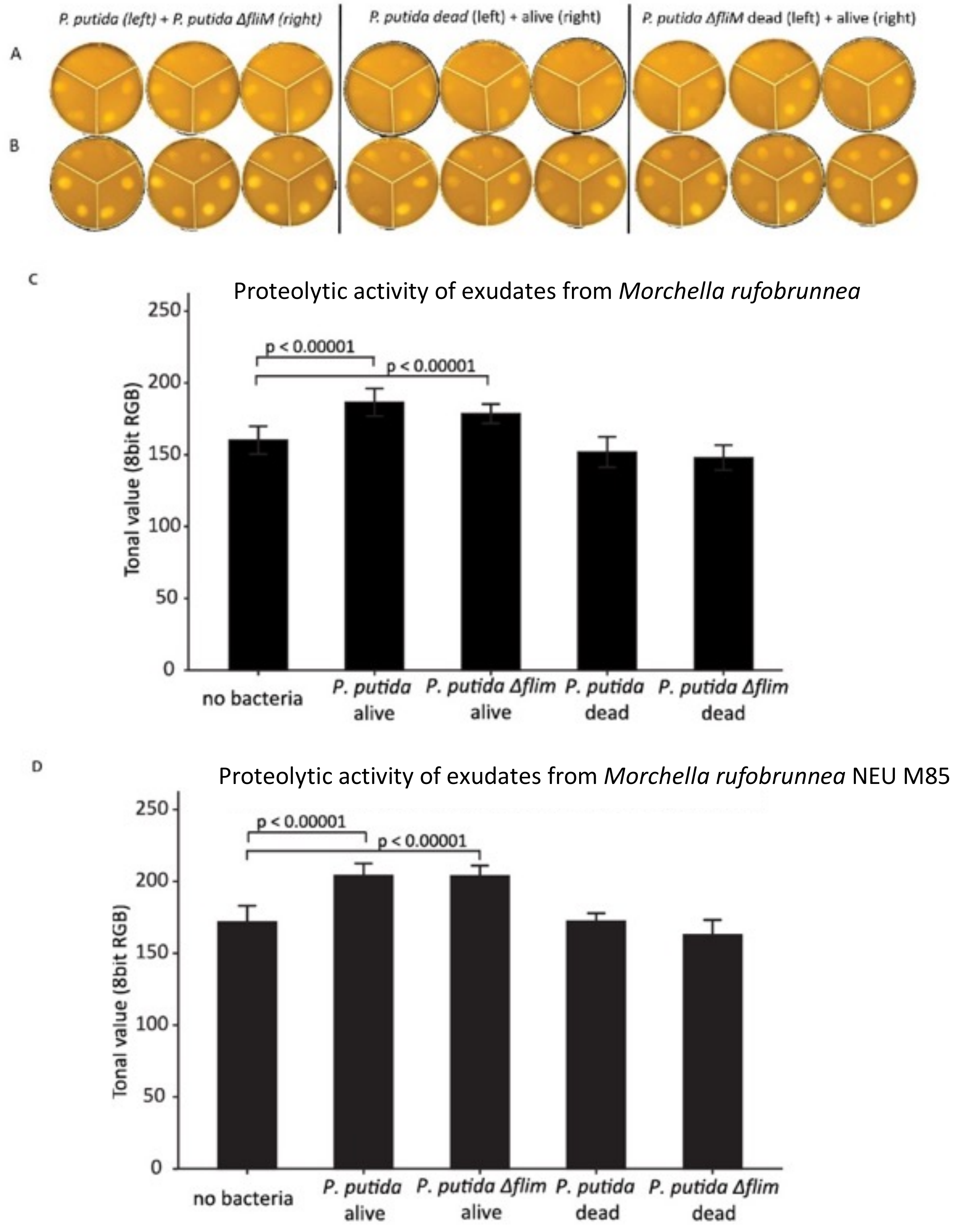
P. putida (left) + P. putida ΔfliM (right)
P. putida dead (left) + alive (right)
P. putida ΔfliM dead (left) + alive (right)
A
B
C
Proteolytic activity of exudates from Morchella rufobrunnea
Tonal value (8bit RGB)
250
200
150
100
50
0
p < 0.00001
p < 0.00001
no bacteria
P. putida alive
P. putida Δflim alive
P. putida dead
P. putida Δflim dead
D
Proteolytic activity of exudates from Morchella rufobrunnea NEU M85
Tonal value (8bit RGB)
250
200
150
100
50
0
p < 0.00001
p < 0.00001
no bacteria
P. putida alive
P. putida Δflim alive
P. putida dead
P. putida Δflim dead

Figure S5. Effect of viable *Pseudomonas putida* cells on fungal exudates extracted from *M. rufobrunnea* and *M. rufobrunnea* NEU 85 measured on skimmed milk medium-agar supplemented with malt (malt/casein). Proteolytic activity of extracted fungal exudates dropped on malt/casein in compartmentalized petri dishes) for *M. rufobrunnae* grown on malt-agar (malt) in mono-culture (**A**) and *M. rufobrunnea* NEU M85 cultivated on malt in monoculture (**B**). The three treatments consisted of a control without bacteria, dead bacteria, and corresponding living bacterial culture (*P.putida* motile or non-motile; Δ*fliM*) as shown in Fig. 2. Technical triplicates were performed for each treatment. Pictures were taken at same light and distance conditions. All images were analyzed with ImageJ by measuring the tonal value means of 40 pixel of a 8 bit RGB images (**C** and **D**). p-values are indicated, when $p < 0.01$ (calculated with a student t-test, two-way hypothesis. The p-values for no bacteria/P. putida alive and no bacteria/*P. putida* Δ*fliM* alive are < 0.00001 and are indicated in each graph. The p-values for *P. putida* alive/dead and *P. putida* Δ*fliM* alive/dead are also < 0.00001, but not indicated in the graphs C: t-value for no bacteria/*P. putida* alive = -7.34879; no bacteria/*P. putida* Δ*fliM* alive = -5.71025; *P. putida* alive/dead = 7.00702; *P. putida* Δ*fliM* alive/dead = 8.31632). D: t-value for no bacteria/*P. putida* alive = -8.51064; no bacteria/*P. putida* Δ*fliM* alive = -8.82218; *P. putida* alive/dead = 8.3513; *P. putida* Δ*fliM* alive/dead = 10.00327).

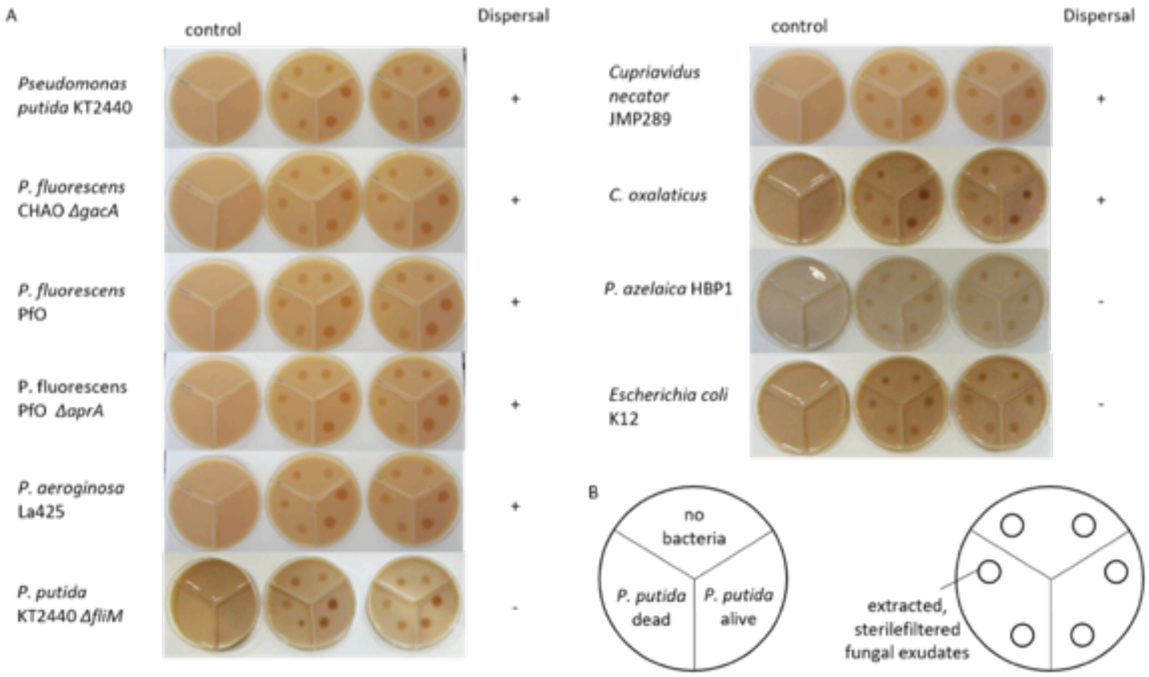

Figure S6. Effect of different bacterial species on proteolytic activity of extracted fungal exudates of *Morchella crassipes* on skimmed milk-agar medium supplemented with malt. Different *Pseudomonas* spp. and other soil bacteria were tested for their ability to enhance extracted fungal exudates (n = 4) (**A**). The ability of the respective bacterium to disperse using fungal highways is indicated as + or – in front of each species. Images of the petri dishes were taken after 24h using a Canon Power Shot SX 230 HS digital camera. A scheme showing the disposition of bacterial treatments in the different compartments is shown in **B**.

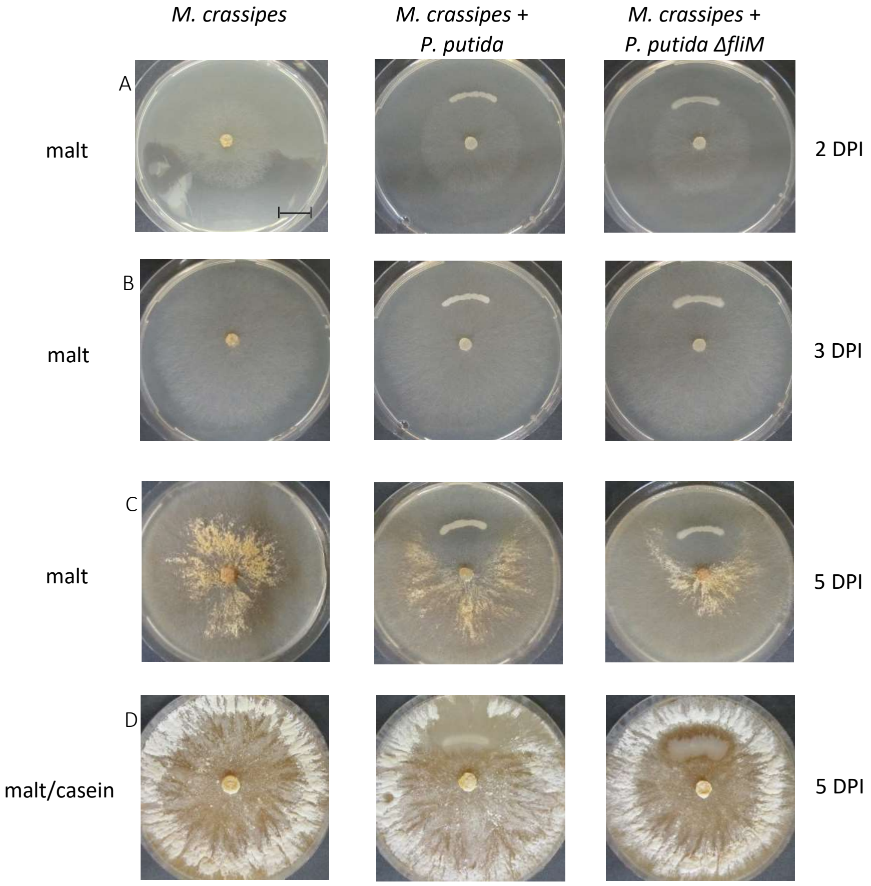

Figure S7. Farming of *Pseudomonas putida* by *Morchella crassipes* observed in malt-agar medium (malt) and comparison with cultures performed on skimmed milk-agar medium supplemented with malt five days post-inoculation (5 DPI). The aspect of cultures containing *M. crassipes* alone (left column) or in co-culture with a motile (middle column) or a non-motile mutant (right column) strain of *P. putida* is shown after two (**A**), three (**B**), and five (**C-D**) days post-inoculation (DPI). Images for the cultures at two and three DPI days for malt/casein are shown as part of Figure 1. Bacterial farming, which is observed macroscopically by the directional formation of sclerotia after five days post-inoculation (DPI) can be observed when co-culturing *M. crassipes* and *P. putida* on malt. Farming occurred in both the motile (middle column) and non-motile mutant (right column) strains. No farming was observed on malt/casein. The scale bar (15 mm) for all images is shown on the bottom of the first image.

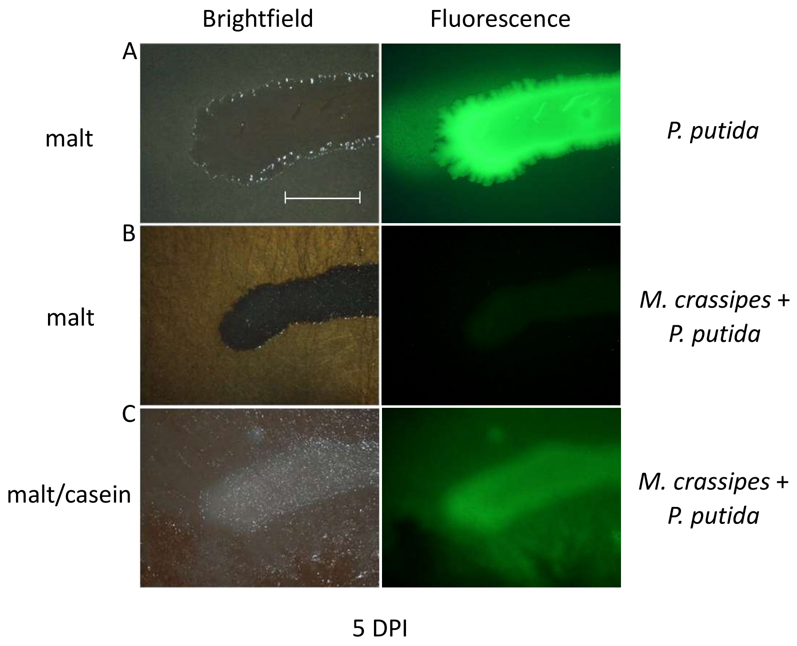

Figure S8. Changes in the emission of fluorescence in the motile GFP-tagged *Pseudomonas putida* KT4220 strain alone or in co-culture with *M. crassipes* in malt-agar medium (malt) and comparison with cultures performed on skimmed milk-agar medium supplemented with malt (malt/casein). A. In a co-culture of *P. putida* with *M. crassipes* on malt fading of the fluorescence is observed for the bacterial inoculum (right column). **B**. When the bacterium is cultured as a monoculture on the same medium, no fading is observed five days after inoculation. **C**. Fluorescence is still visible and no farming is observed when the co-culture is performed on malt/casein. All fluorescence images are shown also in bright field (column to the left). The observations were made on the same petri dishes shown in Figure S7 C,D for the cultures five days post-inoculation (5 DPI). The scales bar shown (A, left) is representative for each image and it corresponds to a length of 2,5 mm.

M. crassipes + *P. putida* on urea

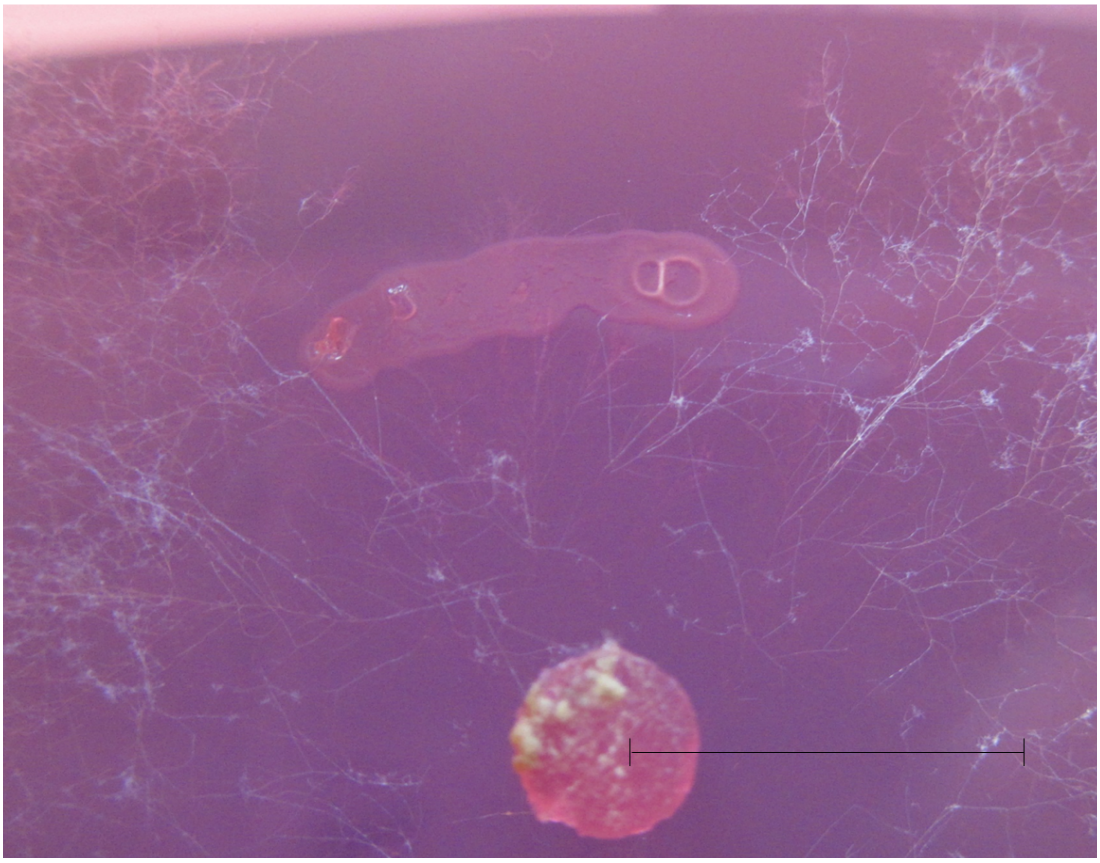

6 DPI

Figure S9. Hyphal growth of *M. crassipes* on urea agar (urea) motile in contact with *Pseudomonas putida* KT4220. The observation of the hyphal growth was made six days post-inoculation (6 DPI). The scales bar shown corresponds to a length of 15 mm.

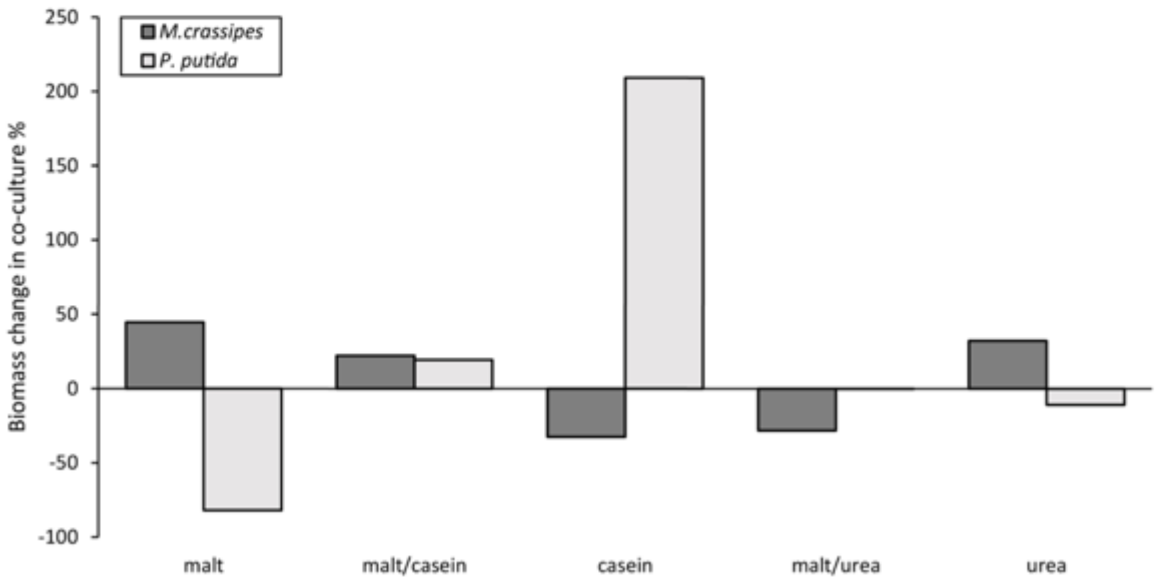

Figure S10. Relative change of biomass in co-cultures for individual partners. The relative biomass for each individual strain was calculated comparing the level of specific FAMEs between mono- and co-culture for each media. The change of biomass in percentage (fungal: blue; bacterial: red) is shown in malt agar (malt), skimmed milk agar supplemented with malt (malt/casein), skimmed milk agar (casein), urea agar supplemented with malt (malt/urea), and urea agar (urea).

Table S1: Annotated functions and orthologous of proteases produced by *P. putida* KT4220 assigned by Kyoto Encyclopaedia of Genes and Genomes.

Protein	Annotated function (Reference Seqence)	Functional ortholog assigned by KEGG
PP 0093	zinc protease	not assigned
PP 0144	insulinase family metalloprotease	not assigned
PP 0199	membrane protease family protein (qmcA)	not assigned
PP 0200	membrane bound peptidase NefD (nfeD)	membrane-bound serine protease (ClpP class) (K07403)
PP 0625	chaperone protein ClpB (clpB)	ATP-dependent Clp protease ATP-binding subunit ClpB (K03695)
PP 0680	ATP-dependent protease-like protein	not assigned
PP 0893	ThiJ/PfpI family protein	protease I [EC:3.2.-.-] (K05520)
PP0940	microcin B17 maturation protease (tldD)	TldD protein (K03568)
PP 0942	regulatory protease (pmbA)	PmbA protein (K03592)
PP 1301	serine endoprotease DegS (degS)	serine protease DegS [EC:3.4.21.-] (K04691)
PP 1321	protease specificity-enhancing factor (sspB;ClpXP)	stringent starvation protein B (K03600)
PP 1430	DegP-like serine endoprotease	serine protease Do [EC:3.4.21.107] (K04771)
PP 1443	lon-I; DNA-binding ATP-dependent protease	Lon-like ATP-dependent protease [EC:3.4.21.-] (K08675)
PP 1598	regulatory intramembrane protein RIP (rseP)	regulator of sigma E protease [EC:3.4.24.-] (K11749)
PP 1719	tail-specific protease Prc (prc)	carboxyl-terminal processing protease [EC:3.4.21.102] (K03797)
PP 1871	protease HtpX (htpX)	heat shock protein HtpX [EC:3.4.24.-] (K03799)
PP1908	putative peptidase	protease IV [EC:3.4.21.-] (K04773)
PP 2206	Peptidase (yegQ)	putative protease [EC:3.4.-.-] (K08303)
PP 2300	ATP-dependent Clp protease proteolytic subunit (clpP)	ATP-dependent Clp protease, protease subunit [EC:3.4.21.92] (K01358)
PP 2301	ATP-dependent Clp protease ATP-binding subunit ClpX (clpX)	ATP-dependent Clp protease ATP-binding subunit ClpX (K03544)
PP 2302	lon-II; DNA-binding ATP-dependent protease	ATP-dependent Lon protease [EC:3.4.21.53] (K01338)
PP2318	acyl-CoA thioesterase I/protease I/lysophospholipase L1 (tesA)	acyl-CoA thioesterase I [EC:3.1.2.- 3.1.1.5] (K10804)
PP 2558	outer membrane efflux protein	outer membrane protein, protease secretion system (K12538)
PP 2559	secretion metalloprotease (hasE)	membrane fusion protein, protease secretion system (K12537)
PP 2560	alkaline protease secretion ABC transporter ATP-binding protein (aprDA)	ATP-binding cassette, subfamily C, bacterial exporter for protease/lipase (K12536)
PP2685	hypothetical protein	putative proteasome-type protease (K07395)
PP 2725	protease PfpI (pfpI)	protease I [EC:3.2.-.-] (K05520)
PP3045	ATP-dependent protease ClpP	not assigned
PP 3267	Clp protease	not assigned
PP 3316	chaperone-associated ATPase	ATP-dependent Clp protease ATP-binding subunit ClpC (K03696)
PP 5604	subtilisin-like serine protease	not assigned
PP 3497	peptidase (ydcP)	putative protease [EC:3.4.-.-] (K08303)
PP 5629	protease/amidase	not assigned
PP 3922	peptidase (sohB)	serine protease SohB [EC:3.4.21.-] (K04774)
PP 3990	FtsH-mediated proteolysis substrate/modulator (yccA)	modulator of FtsH protease (K19416)
PP 4008	ATP-dependent serine protease (clpA)	ATP-dependent Clp protease ATP-binding subunit ClpA (K03694)
PP 4009	ATP-dependent Clp protease adapter protein ClpS (clpS)	ATP-dependent Clp protease adaptor protein ClpS (K06891)
PP 4657	zinc metalloprotease (ypfJ)	uncharacterized protein (K07054)
PP 4718	integral membrane ATP-dependent zinc metallopeptidase (ftsH)	cell division protease FtsH [EC:3.4.24.-] (K03798)
PP 4814	ATP-dependent protease La domain-containing protein	uncharacterized protein (K07157)
PP 4891	integral membrane ATP-dependent zinc metallopeptidase (hflC)	membrane protease subunit HflC [EC:3.4.-.-] (K04087)
PP 4892	integral membrane ATP-dependent zinc metallopeptidase (hflK)	membrane protease subunit HflK [EC:3.4.-.-] (K04088)
PP 4913	hypothetical protein	aspartyl protease family protein (K06985)
PP 4924	subtilase family serine protease	not assigned
PP 5000	ATP-dependent HslVU protease peptidase subunit (hslV)	ATP-dependent HslUV protease, peptidase subunit HslV [EC:3.4.25.2] (K01419)
PP 5001	protease HslVU ATPase subunit (hslU)	ATP-dependent HslUV protease ATP-binding subunit HslU (K03667)
PP 5058	carboxy-terminal-processing protease (ctpA)	carboxyl-terminal processing protease [EC:3.4.21.102] (K03797)
PP 5112	peptidase	zinc protease [EC:3.4.24.-] (K07263)
PP 5113	Zn-dependent peptidase	zinc protease [EC:3.4.24.-] (K07263)
PP 5115	zinc protease	not assigned

Table S2: Media composition for fungal and bacterial strains *if concentration of agar changes, it will be indicated directly in the material and methods

**Compound**	**nutrient broth agar (NA)**	**malt**	**casein**	**malt/ casein**	**urea**	**malt/ urea**
Nutrient broth (Biolife, Italy)	8 g/L	-	-	-	-	-
Malt extract (SIOS, Switzerland)	-	12 g/L	-	12 g/L	-	12 g/L
Skimmed milk powder (Migros, Switzerland)	-	-	50 g/L	50 g/L	-	-
Urea (Fluka, Switzerland)	-	-	-	-	20 g/L	20 g/L
Sodium phosphate monobasic Dihydrate (Fluka, Switzerland)	-	-	-	-	0.91 g/L	0.91 g/L
Phenol red (Siegfried, Switzerland)	-	-	-	-	0.02 g/L	0.02 g/L
Agar (Biolife, Switzerland)*	15 g/L	15 g/L	15 g/L	15 g/L	15 g/L	15 g/L

Table S3: Total organic C/N atomic ratio of medium components used in this study

Medium component	C/N atomic ratio	STD
Malt	55.84	1.73
Skimmed Milk	4.47	0.31
Urea	0.50	0.01

Information (SI)

Sequences of the internal transcribed spacer 1 (partial), 5.8S ribosomal RNA gene (complete sequence) and the internal transcribed spacer 2 (partial sequence) of the *Morchella* strains used in this study. For each strain a summary of the alignment to sequences of the genus *Morchella* in the curated Morchella MLST database from the Westerdijki Institute (http://www.westerdijkinstitute.nl/morchella/) is provided.

>*Morchella rufobrunnea* NEU M85
GGAATCATTACAAAAATCCAACCAAAAAGAGGCGAGGACCGCGGCCGCCTGGTC
TCCCCGGTTAAGAGGCACCCACCCTCAGCCCCCATCTGCGCGCGGCGGGGGATGC
GGGGCCTCCCGTACTGGCCGGGCCGCGGGCCCCTCCGTCTTCACACAAACCCTCT
GTGTACCTTGTCCCCATTGCTTCCCTCGGAACGAGAGCTTCTCCGGGCTGGTCCGG
GTGGCGGCGCCCCGTGGAACGGGCGGCGCAACCCGATTGGCCACGCCTGGCTCG
CTCCTCGCCCGGGGGGAGGAAAACCCAAACAAACCCTTTGCGCGAAAAACGACG
TCTGAACAGAAAAGAAAACAAACTAAAAGTTAAAACTTTCAACAACGGATCTCT
TGGTTCCCACATCGATGAAGAACGCAGCGAAATGCGATAAGTAATGTGAATTGC
AGAATTCAGTGAATCATCGAATCTTTGAACGCACATTGCGCCCTCTGGTATTCCG
GGGGGCATGCCTGTTCGAGCGTCATAAAAACCTCTCCACCCCGGGTTCATGAGAT
CCGGGGCGGGTATTGGCGGTGCGGATCCCTCCCCCCTATACAACGGGACGGAGG
CGCCCACCGCTCAAAGCCATGAAGATAACCCAGCCGAAAGCGCCAGGGCGTCAA
CCGTGGAGTCATGAGGAACCACGCCCCGCGAGGGGTGCCTCCCACTCTGGGCGA
CCGGTGTATGGAAATGCGGGCGTAAAAGCACGGGAAATCCGCGGGAACCGGCGC
CGTCTCTGGCTGCGGCCGTGGATGAT

#	Reference description	Score	Probability	Similarity	Fragments	Overlap%	Direction	Rating
1	SH210000.07FU KM485959 Fungi, Ascomycota, Pezizomycetes, Pezizales, Morchellaceae, Morchella, Morchella rufobrunnea	1245.78	0	100	1	99.619	+/+	*****
2	SH210000.07FU KM588017 Fungi, Ascomycota, Pezizomycetes, Pezizales, Morchellaceae, Morchella, Morchella rufobrunnea	1245.78	0	100	1	99.619	+/+	*****
3	SH210000.07FU DQ355922 Fungi, Ascomycota, Pezizomycetes, Pezizales, Morchellaceae, Morchella, Morchella rufobrunnea	1231.52	0	100	1	98.477	+/+	*****
4	SH210000.07FU JQ723134 Fungi, Ascomycota, Pezizomycetes, Pezizales, Morchellaceae, Morchella, Morchella rufobrunnea	1231.52	0	100	1	98.477	+/+	*****
5	SH210000.07FU JQ723132 Fungi, Ascomycota, Pezizomycetes, Pezizales, Morchellaceae, Morchella, Morchella rufobrunnea	1231.52	0	100	1	98.477	+/+	*****
6	SH210000.07FU JQ723128 Fungi, Ascomycota, Pezizomycetes, Pezizales, Morchellaceae, Morchella, Morchella rufobrunnea	1231.52	0	100	1	98.477	+/+	*****
7	SH210000.07FU JQ723126 Fungi, Ascomycota, Pezizomycetes, Pezizales, Morchellaceae, Morchella, Morchella rufobrunnea	1231.52	0	100	1	98.477	+/+	*****

8	SH210000.07FU JQ723130 Fungi, Ascomycota, Pezizomycetes, Pezizales, Morchellaceae, Morchella, Morchella rufobrunnea	1231.52	0	100	1	98.477	+/+	*****
9	SH210000.07FU JQ723122 Fungi, Ascomycota, Pezizomycetes, Pezizales, Morchellaceae, Morchella, Morchella rufobrunnea	1231.52	0	100	1	98.477	+/+	*****
10	SH210000.07FU JQ723127 Fungi, Ascomycota, Pezizomycetes, Pezizales, Morchellaceae, Morchella, Morchella rufobrunnea	1231.52	0	100	1	98.477	+/+	*****
11	SH210000.07FU JQ723125 Fungi, Ascomycota, Pezizomycetes, Pezizales, Morchellaceae, Morchella, Morchella rufobrunnea	1231.52	0	100	1	98.477	+/+	*****
12	SH210000.07FU JQ723123 Fungi, Ascomycota, Pezizomycetes, Pezizales, Morchellaceae, Morchella, Morchella rufobrunnea	1231.52	0	100	1	98.477	+/+	*****
13	SH210000.07FU JQ723124 Fungi, Ascomycota, Pezizomycetes, Pezizales, Morchellaceae, Morchella, Morchella rufobrunnea	1231.52	0	100	1	98.477	+/+	*****
14	SH210000.07FU JQ691486 Fungi, Ascomycota, Pezizomycetes, Pezizales, Morchellaceae, Morchella, Morchella rufobrunnea	1206.16	0	100	1	96.447	+/+	*****
15	SH210000.07FU JX292976 Fungi, Ascomycota, Pezizomycetes, Pezizales, Morchellaceae, Morchella, Morchella rufobrunnea	1218.84	0	99.872	1	99.365	+/+	****
16	SH210000.07FU JX258671 Fungi, Ascomycota, Pezizomycetes, Pezizales, Morchellaceae, Morchella, Morchella crassipes	1220.42	0	99.746	1	99.619	+/+	****
17	SH210000.07FU JX292975 Fungi, Ascomycota, Pezizomycetes, Pezizales, Morchellaceae, Morchella, Morchella rufobrunnea	1195.06	0	99.745	1	99.365	+/+	****
18	SH210000.07FU JQ723129 Fungi, Ascomycota, Pezizomycetes, Pezizales, Morchellaceae, Morchella, Morchella rufobrunnea	1185.55	0	99.743	1	98.477	+/+	****
19	SH210000.07FU JQ723133 Fungi, Ascomycota, Pezizomycetes, Pezizales, Morchellaceae, Morchella, Morchella rufobrunnea	1202.99	0	99.615	1	98.477	+/+	****
20	SH210000.07FU JQ723131 Fungi, Ascomycota, Pezizomycetes, Pezizales, Morchellaceae, Morchella, Morchella rufobrunnea	1202.99	0	99.615	1	98.477	+/+	****
21	SH210000.07FU DQ355921 Fungi, Ascomycota, Pezizomycetes, Pezizales, Morchellaceae, Morchella, Morchella rufobrunnea	1222.01	0	99.613	1	98.477	+/+	****
22	SH210000.07FU GQ304946 Fungi, Ascomycota, Pezizomycetes, Pezizales,	1155.44	0	99.599	1	94.67	+/+	****

	Morchellaceae, Morchella, Morchella crassipes							
23	SH210000.07FU JX292973 Fungi, Ascomycota, Pezizomycetes, Pezizales, Morchellaceae, Morchella, Morchella rufobrunnea	1191.89	0	99.492	1	99.365	+/+	****
24	SH210000.07FU JX292974 Fungi, Ascomycota, Pezizomycetes, Pezizales, Morchellaceae, Morchella, Morchella rufobrunnea	1109.47	0	98.658	1	94.289	+/+	****
25	MF228802 Fungi, Ascomycota, Pezizomycetes, Pezizales, Morchellaceae, Morchella, Morchella anatolica	828.935	0	94.505	1	91.244	+/+	***
26	MF228801 Fungi, Ascomycota, Pezizomycetes, Pezizales, Morchellaceae, Morchella, Morchella anatolica	873.314	0	93.79	1	98.985	+/+	***
27	SH525013.07FU KJ802105 Fungi, Ascomycota, Pezizomycetes, Pezizales, Morchellaceae, Morchella, Morchella anatolica	838.445	0	93.611	1	96.193	+/+	***
28	SH525013.07FU KM587974 Fungi, Ascomycota, Pezizomycetes, Pezizales, Morchellaceae, Morchella, Morchella anatolica	884.409	0	93.409	1	98.985	+/+	***

>*Morchella rufobrunnea*
GGTCTCCCCGGTTAAGAGGCACCCACCCTCAGCCCCCATCTGCGCGCGGCGGGGG
ATGCGGGGCCTCCCGTACTGGCCGGGCCGCGGGCCCCTCCGTCTTCACACAAACC
CTCTGTGTACCTTGTCCCCATTGCTTCCCTCGGAACGAGAGCTTCTCCGGGCTGGT
CCGGGTGGCGGCGCCCCGTGGAACGGGCGGCGCAACCCGATTGGCCACGCCTGG
CTCGCTCCTCGCCCGGGGGGAGGAAAACCCAAACAAACCCTTTGCGCGAAAAAC
GACGTCTGAACAGAAAAGAAAACAAACTAAAAGTTAAAACTTTCAACAACGGAT
CTCTTGGTTCCCACATCGATGAAGAACGCAGCGAAATGCGATAAGTAATGTGAAT
TGCAGAATTCAGTGAATCATCGAATCTTTGAACGCACATTGCGCCCTCTGGTATT
CCGGGGGGCATGCCTGTTCGAGCGTCATAAAAACCTCTCCACCCCGGGTTCATGA
GATCCGAGGGCGGGT

#	Reference description	Score	Probability	Similarity	Fragments	Overlap%	Direction	Rating
1	SH210000.07FU KM485959 Fungi, Ascomycota, Pezizomycetes, Pezizales, Morchellaceae, Morchella, Morchella rufobrunnea	797.338	0	99.801	1	96.724	+/+	****
2	SH210000.07FU DQ355921 Fungi, Ascomycota, Pezizomycetes, Pezizales, Morchellaceae, Morchella, Morchella rufobrunnea	797.338	0	99.801	1	96.724	+/+	****
3	SH210000.07FU DQ355922 Fungi, Ascomycota, Pezizomycetes, Pezizales, Morchellaceae, Morchella, Morchella rufobrunnea	797.338	0	99.801	1	96.724	+/+	****
4	SH210000.07FU JQ723134 Fungi, Ascomycota, Pezizomycetes, Pezizales, Morchellaceae, Morchella, Morchella rufobrunnea	797.338	0	99.801	1	96.724	+/+	****
5	SH210000.07FU JQ723130 Fungi, Ascomycota, Pezizomycetes, Pezizales, Morchellaceae, Morchella, Morchella rufobrunnea	797.338	0	99.801	1	96.724	+/+	****
6	SH210000.07FU JQ723128 Fungi, Ascomycota, Pezizomycetes, Pezizales, Morchellaceae, Morchella, Morchella rufobrunnea	797.338	0	99.801	1	96.724	+/+	****
7	SH210000.07FU JQ723126 Fungi, Ascomycota, Pezizomycetes, Pezizales, Morchellaceae, Morchella, Morchella rufobrunnea	797.338	0	99.801	1	96.724	+/+	****
8	SH210000.07FU JQ723124 Fungi, Ascomycota, Pezizomycetes, Pezizales, Morchellaceae, Morchella, Morchella rufobrunnea	797.338	0	99.801	1	96.724	+/+	****
9	SH210000.07FU JQ723132 Fungi, Ascomycota, Pezizomycetes, Pezizales, Morchellaceae, Morchella, Morchella rufobrunnea	797.338	0	99.801	1	96.724	+/+	****
10	SH210000.07FU JQ723129 Fungi, Ascomycota, Pezizomycetes, Pezizales, Morchellaceae, Morchella, Morchella rufobrunnea	797.338	0	99.801	1	96.724	+/+	****
11	SH210000.07FU JQ723127 Fungi, Ascomycota, Pezizomycetes, Pezizales, Morchellaceae, Morchella, Morchella rufobrunnea	797.338	0	99.801	1	96.724	+/+	****

12	SH210000.07FU JQ723125 Fungi, Ascomycota, Pezizomycetes, Pezizales, Morchellaceae, Morchella, Morchella rufobrunnea	797.338	0	99.801	1	96.724	+/+	****
13	SH210000.07FU JQ723123 Fungi, Ascomycota, Pezizomycetes, Pezizales, Morchellaceae, Morchella, Morchella rufobrunnea	797.338	0	99.801	1	96.724	+/+	****
14	SH210000.07FU JQ723122 Fungi, Ascomycota, Pezizomycetes, Pezizales, Morchellaceae, Morchella, Morchella rufobrunnea	797.338	0	99.801	1	96.724	+/+	****
15	SH210000.07FU JQ691486 Fungi, Ascomycota, Pezizomycetes, Pezizales, Morchellaceae, Morchella, Morchella rufobrunnea	797.338	0	99.801	1	96.724	+/+	****
16	SH210000.07FU JX292976 Fungi, Ascomycota, Pezizomycetes, Pezizales, Morchellaceae, Morchella, Morchella rufobrunnea	797.338	0	99.801	1	96.724	+/+	****
17	SH210000.07FU KM588017 Fungi, Ascomycota, Pezizomycetes, Pezizales, Morchellaceae, Morchella, Morchella rufobrunnea	797.338	0	99.801	1	96.724	+/+	****
18	SH210000.07FU JX258671 Fungi, Ascomycota, Pezizomycetes, Pezizales, Morchellaceae, Morchella, Morchella crassipes	794.155	0	99.602	1	96.724	+/+	****
19	SH210000.07FU JX292975 Fungi, Ascomycota, Pezizomycetes, Pezizales, Morchellaceae, Morchella, Morchella rufobrunnea	773.465	0	99.602	1	96.724	+/+	****
20	SH210000.07FU JQ723131 Fungi, Ascomycota, Pezizomycetes, Pezizales, Morchellaceae, Morchella, Morchella rufobrunnea	768.691	0	99.208	1	96.724	+/+	****
21	SH210000.07FU JQ723133 Fungi, Ascomycota, Pezizomycetes, Pezizales, Morchellaceae, Morchella, Morchella rufobrunnea	768.691	0	99.208	1	96.724	+/+	****
22	SH210000.07FU GQ304946 Fungi, Ascomycota, Pezizomycetes, Pezizales, Morchellaceae, Morchella, Morchella crassipes	768.691	0	99.208	1	96.724	+/+	****
23	SH210000.07FU JX292973 Fungi, Ascomycota, Pezizomycetes, Pezizales, Morchellaceae, Morchella, Morchella rufobrunnea	746.41	0	99.012	1	96.724	+/+	****
24	SH210000.07FU JX292974 Fungi, Ascomycota, Pezizomycetes, Pezizales, Morchellaceae, Morchella, Morchella rufobrunnea	730.494	0	98.016	1	96.724	+/+	****
25	MF228801 Fungi, Ascomycota, Pezizomycetes, Pezizales, Morchellaceae, Morchella, Morchella anatolica	555.428	1.17917E-156	93.927	1	94.027	+/+	**
26	MF228802 Fungi, Ascomycota, Pezizomycetes, Pezizales, Morchellaceae,	555.428	1.17917E-156	93.927	1	94.027	+/+	**

	Morchella, Morchella anatolica							
27	SH525013.07FU KJ802105 Fungi, Ascomycota, Pezizomycetes, Pezizales, Morchellaceae, Morchella, Morchella anatolica	555.428	1.17917E-156	93.927	1	94.027	+/+	**
28	SH525013.07FU KM587974 Fungi, Ascomycota, Pezizomycetes, Pezizales, Morchellaceae, Morchella, Morchella anatolica	569.752	5.75105E-161	93.522	1	94.027	+/+	**

Morchella crassipes (JX258671.1)

>JX258671.1 Morchella crassipes strain NEUF 387 18S ribosomal RNA gene, partial sequence; internal transcribed spacer 1 and 5.8S ribosomal RNA gene, complete sequence; and internal transcribed spacer 2, partial sequence

CATCCTCCCCACTTATACAGATAAACCCACACTTAACATATTATAGTGGTTTTTTT
TTTTGTTTTGTTGTGTTGTTTATTTTTGAAGGGTGAAAGTCGTAACAAGGTTTCCG
TAGGTGAACCTGCGGAAGGATCATTACAAAAATCCAACCAAAAAGAGGCGAGGA
CCGCGGCCGCCTGGTCTCCCCGGTTAAGAGGCACCCACCCTCAGCCCCCATCTGC
GCGCGGCGGGGGATGCGGGGCCTCCCGTACTGGCCGGGCCGCGGGCCCCTCCGT
CTTCACACAAACCCTCTGTGTACCTTGTCCCCATTGCTTCCCTCGGAACGAGAGCT
TCTCCGGGCTGGTCCGGGTGGCGGCGCCCCGTGGAACGGGCGGCGCAACCCGAT
TGGCCACGCCTGGCTCGCTCCTCGCCTGGGGGGAGGAAAACCCAAACAAACCCTT
TGCGCGAAAAACGACGTCTGAACAGAAAAGAAAACAAACTAAAAGTTAAAACTT
TCAACAACGGATCTCTTGGTTCCCACATCGATGAAGAACGCAGCGAAATGCGATA
AGTAATGTGAATTGCAGAATTCAGTGAATCATCGAATCTTTGAACGCACATTGCG
CCCTCTGGTATTCCGGGGGGCATGCCTGTTCGAGCGTCATAAAAACCTCTCCACC
CCGGGTTCATGAGATCCGGGGCGGGTATTGGCGGTGCGGATCCCTCCCCCCTATA
CAACGGGACGGAGGCGCCCACCGCTCAAAGCCATGAAGATAACCCAGCCGAAAG
CGCCAGGGCGTCAACCGTGGAGTCATGAGGAACCACGCCCCGCGAGGGGTGCCT
CCCACTCTGGGCGACCCGGTGTATGGAAATGCGGGCGTAAAAGCACGGGAAATC
CGCGGGAACCGGCGCCGTCTCTGGCTGCGGCCGTGGATGATCCCCGTGGGGGGCT
AGATACCCAATCACATTCAAGTTTTGACCTCGATCAGTAGGGATACCCGAGTCAC

#	Reference description	Score	Probability	Similarity	Fragments	Overlap%	Direction	Rating
1	SH210000.07FU JX258671 Fungi, Ascomycota, Pezizomycetes, Pezizales, Morchellaceae, Morchella, Morchella crassipes	1601.33	0	100	1	90.376	+/+	*****
2	SH210000.07FU KM588017 Fungi, Ascomycota, Pezizomycetes, Pezizales, Morchellaceae, Morchella, Morchella rufobrunnea	1369.32	0	99.767	1	78.827	+/+	***
3	SH210000.07FU KM485959 Fungi, Ascomycota, Pezizomycetes, Pezizales, Morchellaceae, Morchella, Morchella rufobrunnea	1351.47	0	99.764	1	77.819	+/+	***
4	SH210000.07FU DQ355922 Fungi, Ascomycota, Pezizomycetes, Pezizales, Morchellaceae, Morchella, Morchella rufobrunnea	1309.29	0	99.757	1	75.435	+/+	***
5	SH210000.07FU JQ723134 Fungi, Ascomycota, Pezizomycetes, Pezizales, Morchellaceae, Morchella, Morchella rufobrunnea	1294.68	0	99.754	1	74.61	+/+	***
6	SH210000.07FU JQ723132 Fungi, Ascomycota, Pezizomycetes, Pezizales, Morchellaceae, Morchella, Morchella rufobrunnea	1294.68	0	99.754	1	74.61	+/+	***
7	SH210000.07FU JQ723128 Fungi, Ascomycota, Pezizomycetes, Pezizales, Morchellaceae, Morchella, Morchella rufobrunnea	1294.68	0	99.754	1	74.61	+/+	***
8	SH210000.07FU JQ723126 Fungi, Ascomycota, Pezizomycetes, Pezizales,	1294.68	0	99.754	1	74.61	+/+	***

	Morchellaceae, Morchella, Morchella rufobrunnea							
9	SH210000.07FU JQ723124 Fungi, Ascomycota, Pezizomycetes, Pezizales, Morchellaceae, Morchella, Morchella rufobrunnea	1294.68	0	99.754	1	74.61	+/+	***
10	SH210000.07FU JQ723130 Fungi, Ascomycota, Pezizomycetes, Pezizales, Morchellaceae, Morchella, Morchella rufobrunnea	1294.68	0	99.754	1	74.61	+/+	***
11	SH210000.07FU JQ723127 Fungi, Ascomycota, Pezizomycetes, Pezizales, Morchellaceae, Morchella, Morchella rufobrunnea	1294.68	0	99.754	1	74.61	+/+	***
12	SH210000.07FU JQ723125 Fungi, Ascomycota, Pezizomycetes, Pezizales, Morchellaceae, Morchella, Morchella rufobrunnea	1294.68	0	99.754	1	74.61	+/+	***
13	SH210000.07FU JQ723123 Fungi, Ascomycota, Pezizomycetes, Pezizales, Morchellaceae, Morchella, Morchella rufobrunnea	1294.68	0	99.754	1	74.61	+/+	***
14	SH210000.07FU JQ723122 Fungi, Ascomycota, Pezizomycetes, Pezizales, Morchellaceae, Morchella, Morchella rufobrunnea	1294.68	0	99.754	1	74.61	+/+	***
15	SH210000.07FU JQ691486 Fungi, Ascomycota, Pezizomycetes, Pezizales, Morchellaceae, Morchella, Morchella rufobrunnea	1268.72	0	99.749	1	73.144	+/+	***
16	SH210000.07FU JX292976 Fungi, Ascomycota, Pezizomycetes, Pezizales, Morchellaceae, Morchella, Morchella rufobrunnea	1296.31	0	99.639	1	76.077	+/+	***
17	SH210000.07FU JX292975 Fungi, Ascomycota, Pezizomycetes, Pezizales, Morchellaceae, Morchella, Morchella rufobrunnea	1271.97	0	99.518	1	76.077	+/+	***
18	SH210000.07FU JQ723129 Fungi, Ascomycota, Pezizomycetes, Pezizales, Morchellaceae, Morchella, Morchella rufobrunnea	1247.63	0	99.509	1	74.61	+/+	***
19	SH210000.07FU DQ355921 Fungi, Ascomycota, Pezizomycetes, Pezizales, Morchellaceae, Morchella, Morchella rufobrunnea	1299.55	0	99.392	1	75.435	+/+	***
20	SH210000.07FU JQ723131 Fungi, Ascomycota, Pezizomycetes, Pezizales, Morchellaceae, Morchella, Morchella rufobrunnea	1265.48	0	99.388	1	74.61	+/+	***
21	SH210000.07FU JQ723133 Fungi, Ascomycota, Pezizomycetes, Pezizales, Morchellaceae, Morchella, Morchella rufobrunnea	1265.48	0	99.388	1	74.61	+/+	***
22	SH210000.07FU GQ304946 Fungi, Ascomycota, Pezizomycetes, Pezizales, Morchellaceae, Morchella, Morchella crassipes	1231.41	0	99.372	1	72.686	+/+	***

23	SH210000.07FU JX292973 Fungi, Ascomycota, Pezizomycetes, Pezizales, Morchellaceae, Morchella, Morchella rufobrunnea	1268.72	0	99.281	1	76.077	+/+	***
24	SH210000.07FU JX292974 Fungi, Ascomycota, Pezizomycetes, Pezizales, Morchellaceae, Morchella, Morchella rufobrunnea	1184.36	0	98.485	1	72.411	+/+	***
25	MF228802 Fungi, Ascomycota, Pezizomycetes, Pezizales, Morchellaceae, Morchella, Morchella anatolica	858.239	0	92.526	1	69.753	+/+	**
26	MF228801 Fungi, Ascomycota, Pezizomycetes, Pezizales, Morchellaceae, Morchella, Morchella anatolica	921.515	0	92.099	1	76.352	+/+	**
27	SH525013.07FU KM587974 Fungi, Ascomycota, Pezizomycetes, Pezizales, Morchellaceae, Morchella, Morchella anatolica	971.812	0	91.995	1	79.927	+/+	**
28	SH525013.07FU KJ802105 Fungi, Ascomycota, Pezizomycetes, Pezizales, Morchellaceae, Morchella, Morchella anatolica	884.198	0	91.879	1	74.244	+/+	**
